# Biochemical characterisation of Mer3 helicase interactions and the protection of meiotic recombination intermediates

**DOI:** 10.1093/nar/gkad175

**Published:** 2023-03-21

**Authors:** Veronika Altmannova, Magdalena Firlej, Franziska Müller, Petra Janning, Rahel Rauleder, Dorota Rousova, Andreas Schäffler, Tanja Bange, John R Weir

**Affiliations:** Friedrich Miescher Laboratory of the Max Planck Society, Max-Planck-Ring 9, 72076 Tübingen, Germany; Friedrich Miescher Laboratory of the Max Planck Society, Max-Planck-Ring 9, 72076 Tübingen, Germany; Department of Mechanistic Cell Biology, Max Planck Institute of Molecular Physiology, Otto-Hahn-Str. 11, 44227, Dortmund, Germany; Department of Chemical Biology, Max Planck Institute of Molecular Physiology, Otto-Hahn-Str. 11, 44227, Dortmund, Germany; Friedrich Miescher Laboratory of the Max Planck Society, Max-Planck-Ring 9, 72076 Tübingen, Germany; Friedrich Miescher Laboratory of the Max Planck Society, Max-Planck-Ring 9, 72076 Tübingen, Germany; Friedrich Miescher Laboratory of the Max Planck Society, Max-Planck-Ring 9, 72076 Tübingen, Germany; Institute of Medical Psychology, Faculty of Medicine, LMU Munich, Germany; Friedrich Miescher Laboratory of the Max Planck Society, Max-Planck-Ring 9, 72076 Tübingen, Germany

## Abstract

Crossing over between homologs is critical for the stable segregation of chromosomes during the first meiotic division. *Saccharomyces cerevisiae* Mer3 (HFM1 in mammals) is a SF2 helicase and member of the ZMM group of proteins, that facilitates the formation of the majority of crossovers during meiosis. Here, we describe the structural organisation of Mer3 and using AlphaFold modelling and XL-MS we further characterise the previously described interaction with Mlh1–Mlh2. We find that Mer3 also forms a previously undescribed complex with the recombination regulating factors Top3 and Rmi1 and that this interaction is competitive with Sgs1^BLM^ helicase. Using *in vitro* reconstituted D-loop assays we show that Mer3 inhibits the anti-recombination activity of Sgs1 helicase, but only in the presence of Dmc1. Thus we provide a mechanism whereby Mer3 interacts with a network of proteins to protect Dmc1 derived D-loops from dissolution.

## INTRODUCTION

Most sexually reproducing organisms utilise meiotic recombination to both link homologous chromosomes during meiosis I, and to generate genetic diversity among their gametes and subsequent progeny. Recombination is initiated by the controlled generation of double-stranded DNA breaks at the onset of prophase I (1,2). While the initial processing of these breaks is analogous to DNA repair in the soma, two important modulations occur in the germline to generate the required crossovers (COs) between homologous chromosomes. Firstly, repair is biased to using the homologous chromosome as a donor rather than the sister (3)), secondly the repair intermediates must be protected from disassembly by anti-CO factors, that prevent the formation of inter-homolog COs and a resulting loss of heterozygosity (LoH) in the soma (4).

One such ‘anti-crossover’ factor is the *Saccharomyces cerevisiae* helicase Sgs1, which is functionally orthologous to the Bloom-syndrome helicase BLM(5). Sgs1 performs its activities in a complex with the type IA topoisomerase Top3, and the OB-fold accessory factor Rmi1 (5–8). STR complex combines helicase and decatenase activities to displace strand invasion intermediates (9,10), and dissolve double-Holliday junctions (dHJs) (11), and thus contributes to genome stability in mitotically dividing cells. During meiosis Sgs1 and STR activity is, somewhat counter intuitively, required for normal crossover formation (12–16). However, Sgs1 is not always active during meiosis, and its activity is instead modulated through CDK phosphorylation (17).

**Figure 1.**
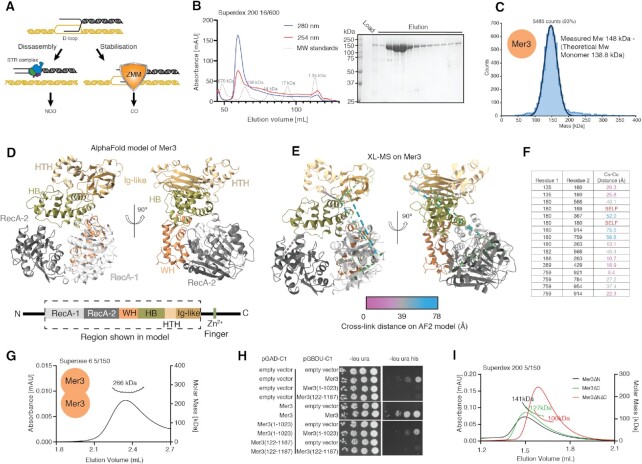
Physical and Structural Analysis of Mer3 helicase. (**A**) Cartoon representation of two possible outcomes for D-loop DNA repair intermediates during meiosis in budding yeast. Either the D-loop is disassembled by the Sgs1 helicase (in the Sgs1/Top3/Rmi1–STR – complex) or the D-loop is captured and stabilised by members of the ZMM group of proteins. (**B**) Purification of the meiosis-specific helicase Mer3. Upper panel: size exclusion chromatography (Superdex 200 16/600) profile of recombinant Mer3; red trace = absorbance at 254 nm, blue trace absorbance at 280 nm, grey trace = SEC standards. Lower panel: coomassie stained SDS-PAGE gel of the SEC column elution. (**C**) Molecular mass determination of Mer3 using Mass Photometry (30 nM protein concentration), showing that we only detect Mer3 monomers at this concentration. (**D**) AlphaFold2 model of *S. cerevisiae* Mer3 from the publicly available database (https://www.alphafold.ebi.ac.uk/). Domains are assigned based on the Brr2 helicase helicase structure (55). (**E**) Cross-links (DSBU cross-linker) modelled onto the Mer3 AF2 structure, and coloured according to distance using XMAS (45), with purple cross-links generally consistent with the model, and blue cross-links in violation. (**F**) List of Mer3 cross-links indicated the residues cross-links and the range of distances (cross-links of <30 Å can be considered compatible with the model). The self cross-links are highlighted that can only be compatible with a higher order complex. (**G**) SEC-MALS on Mer3 (10 μM injection concentration) showing that Mer3 can form homodimers at higher concentrations. (**H**) Yeast-two-hybrid analysis of Mer3 self interaction. Samples were grown on indicated plates for four days prior to imaging. (**I**) SEC-MALS (10 μM injection concentration) on three purified Mer3 truncation constructs Mer3ΔN (122–1187), Mer3ΔC (1–1023) and Mer3ΔNΔC (122–1023). The theoretical molecular masses for the monomers are: Mer3ΔN = 126 kDa; Mer3ΔC = 121 kDa, Mer3ΔNΔC = 107 kDa.

In budding yeast, a group of proteins was identified that, in general, promoted CO formation, and was collectively termed ‘ZMM’ (18) The *S. cerevisiae* group of ZMM proteins consists of Zip1, Zip2, Zip3, Zip4, Spo16, the Mer3 helicase, and the Msh4–Msh5 heterodimer (19,20). The ZMMs are involved in the formation and stabilisation of single-end invasion (SEI) intermediates (18,21); ZMM mutants show a decrease in the formation of SEI and dHJ intermediates. In absence of ZMMs spore viability is decreased as well as the number of COs (18,22). ZMM proteins were also presumed to counteract the anti-crossover activity of Sgs1 helicase (15,22) (Figure 1A).

Mer3 helicase is well conserved, with functional orthologs being found in other fungi (23), plants (24–26) and mammals (27), where it is also required for human fertility (28). *In vitro* studies on Mer3 showed that it is an active ATPase with strand separation activity working in 3′ to 5′ direction (29) and that it might preferentially recognize Holliday junctions, however, it also recognizes other DNA structures (30,31). Further *in vitro* works demonstrated that Mer3 promoted a heteroduplex extension by Rad51, that is, it enlarged and stabilised D-loops (32). It was shown that Mer3, in addition to other ZMM proteins, acts to protect nascent CO-designated recombination intermediates from disassembly by Sgs1 (22).

While the mechanisms for chromosomal recruitment of Mer3 are unclear, it does appear that Mer3 is recruited early in the DNA repair pathway (23,33)*. In vivo* Mer3 ATPase deficient mutants (mer3G166D and mer3K167A) show mild spore viability defects whereas in *mer3Δ* strain spore viability is strongly compromised (30,31). This observation hints at the possibility that Mer3 may contribute to promoting crossover formation through protein–protein interactions. To date, Mer3 has been reported to interact with only a few proteins involved in meiotic recombination such as the helicase Pif1, replication factor Rfc1 (34), and the MutLβ complex (Mlh1/Mlh2) (31). The interaction between Mer3 and MutLβ was shown to occur via the Ig-like domain of Mer3. Impairing the ability of Mer3 to bind to Mlh2 leads to an increase in the length of gene conversion tracts, both in COs and in NCOs (31).

Here we used biochemical and structural approaches to describe the activity, oligomeric status and structural organisation of Mer3. We also further characterised the details of the Mer3 interaction with the Mlh1/Mlh2 complex. The search for novel Mer3 interactors has led us to discover that Mer3 forms a complex with Top3 and Rmi1 and that this interaction is compatible with Mlh1/Mlh2 binding. Interestingly, Mer3 is able to disrupt Top3-Rmi1 binding to Sgs1^BLM^ helicase in a phospho-dependent manner. We further show that Mer3 inhibits D-loop disassembly mediated by the Sgs1–Top3–Rmi1 complex in the presence of Dmc1 recombinase thus revealing a novel mechanism for the protection of DNA repair intermediates and thus promotion of crossover formation during meiosis I.

## MATERIALS AND METHODS

### Cloning

Sequences of *S. cerevisiae* MER3, MLH1, MLH2, TOP3, RMI1 and SGS1 were derived from SK1 strain genomic DNA. Due to the presence of an intron in *MER3*, this was amplified as two separate fragments and Gibson assembled. Plasmids used for protein expression were cloned as previously described from the InteBac (35) and biGBac vector suites (36). Baculovirus was generated via the EMBacY cells, part of the MultiBac expression system (37). For a full list of plasmids please see Supplementary Table S1.

### Protein expression

Mer3, Mlh1, Mlh2, Top3, Rmi1, full-length Sgs1 and Rad54 were produced in the Hi-5 cell line derived from the cabbage looper *Trichoplusiani*. Bacmids for expression were produced in EmBacY cells and subsequently used to transfect Sf9 cells to produce baculovirus. Amplified baculovirus was used to infect Hi-5 cells in 1:100 dilution prior to 72-h cultivation and harvest. Cells were washed twice with 1× PBS, frozen in liquid nitrogen and the pellets were stored at -80°C.

### Protein purification

Mer3 was produced as a C-terminal Twin-StrepII tag in Hi5 insect cells using the same expression conditions as described above.To purify Mer3-Strep, cells were resuspended in lysis buffer (50 mM HEPES pH 6.8, 300 mM NaCl, 5% glycerol, 0.1% Triton-X 100, 1 mM MgCl_2_, 5 mM beta-mercaptoethanol). Resuspended cells were lysed using an EmulsiFlex C3 (Avestin) in presence of Serva Protease-Inhibitor Mix and Benzonase before clearance at 130 000g at 4°C for 1 h. Cleared lysate was applied on a 5 ml Strep-Tactin®XT column (IBA) and extensively washed with lysis buffer. Mer3 constructs were eluted with a lysis buffer containing 50 mM Biotin. Eluted protein was passed through a HiTrap Heparin HP affinity column (Cytiva) pre-equilibrated with the loading buffer (50 mM HEPES pH 6.8, 300 mM NaCl, 5% glycerol, 1 mM MgCl_2_, 5 mM β-mercaptoethanol). The proteins were eluted by increasing salt gradient to 1 M NaCl. Protein-containing elution fractions were concentrated on Vivaspin 15R, 30 000 MWCO Hydrosart concentrators. The concentrated eluent was loaded on a Superdex 200 16/600 pre-equilibrated in SEC buffer (30 mM MES pH 6.5, 300 mM NaCl, 5% glycerol, 1 mM MgCl_2_, 1 mM TCEP). Purified protein was concentrated using Vivaspin 15R, 30 000 MWCO Hydrosart concentrators. Dephosphorylated Mer3 (denoted as ‘Mer3-λ’) was prepared using a similar procedure which included lambda-phosphatase (New England Biolabs, P0753) treatment of concentrated Mer3 before loading on the size-exclusion column. To prepare phosphorylated Mer3 (denoted as ‘Mer3-P’), 100 nM okadaic acid was added to the Hi5 cells for the last 3 h before harvesting. Protein was then purified using the same protocol as described above. Mer3-K167A helicase-dead mutant and Mer3 fragments were purified using the same protocol as wild type Mer3 protein.

To purify the MBP-tagged Mlh1-Mlh2 complex, N-terminal 6xHis-MBP tagged Mlh1 and Mlh2 were cloned into the pBIG1a vector and expressed in Hi5 insect cells using the same expression conditions as described above. The cell pellet was resuspended in lysis buffer (50 mM HEPES pH 6.8, 300 mM NaCl, 5% glycerol, 0.1% Triton X-100, 1 mM MgCl_2_, 5 mM β-mercaptoethanol). Resuspended cells were lysed using an EmulsiFlex C3 (Avestin) in presence of Serva Protease-Inhibitor Mix and Benzonase before clearance at 130 000g at 4°C for 1 h. Cleared lysate was applied on a 5 ml MBP-trap column (Cytiva) and extensively washed with lysis buffer. Mlh1/Mlh2 complex was eluted with a lysis buffer containing 1 mM maltose. Eluted protein was passed through a HiTrap Heparin HP affinity column (Cytiva) pre-equilibrated with the loading buffer (50 mM HEPES pH 6.8, 300 mM NaCl, 5% glycerol, 1 mM MgCl_2_, 5 mM βa-mercaptoethanol). The proteins were eluted by increasing salt gradient to 1 M NaCl. Protein-containing elution fractions were concentrated on Vivaspin 15R, 30 000 MWCO Hydrosart concentrators. The concentrated eluent was loaded on a Superdex 200 16/600 pre-equilibrated in SEC buffer (30 mM HEPES 6.8, 300 mM NaCl, 5% glycerol, 1 mM MgCl_2_, 1 mM TCEP). Purified protein was concentrated using Vivaspin 15R, 30 000 MWCO Hydrosart concentrators and stored at -80°C in small aliquots.

To purify Strep/His-tagged Mlh1–Mlh2 complex, N-terminal 6xHis tagged-Mlh1 and N-terminal Twin-StrepII tagged Mlh2 were cloned into the pBIG1a vector and expressed in Hi5 insect cells using the same expression conditions as described above. The cell pellet was resuspended in the lysis buffer (50 mM HEPES pH 7.5, 300 mM NaCl, 5% glycerol, 0.01% NP40, 5 mM β-mercaptoethanol, AEBSF, Serva protease inhibitor cocktail). Resuspended cells were lysed by sonication before clearance at 35 000 rpm at 4°C for 1 h. Cleared lysate was loaded on a 5 ml Strep-Tactin XT 4Flow column (IBA) followed by the first wash using 25 ml of H buffer (20 mM HEPES pH 7.5, 5% glycerol, 0.01% NP40, 1 mM β-mercaptoethanol) containing 500 mM NaCl and the second wash with H buffer containing 150 mM NaCl. Mlh1–Mlh2 complex was eluted with a 50-ml gradient of H buffer containing 150 mM NaCl and 50 mM biotin. Partially purified protein was further loaded onto a 5 ml HiTrap Heparin HP affinity column (Cytiva) pre-equilibrated in H buffer containing 150 mM NaCl and eluted with an increasing salt gradient to 1 M NaCl. The fractions containing Mlh1-Mlh2 complex were then concentrated on a 50 kDa MWCO Amicon concentrator and applied onto a Superose 6 10/300 column (Cytiva) pre-equilibrated in SEC buffer (20 mM HEPES pH 7.5, 300 mM NaCl, 5% glycerol, 1 mM β-mercaptoethanol, 1 mM TCEP). The fractions containing Mlh1–Mlh2 were concentrated on a 50 kDa MWCO Amicon concentrator and stored at -80°C in small aliquots.

Top3 was produced as an N-terminal 6xHis tag in Hi5 insect cells using the same expression conditions as described above. The cell pellet was resuspended in the lysis buffer (50 mM HEPES pH 7.5, 300 mM NaCl, 5% glycerol, 0.01% NP40, 5 mM β-mercaptoethanol, AEBSF). Resuspended cells were lysed by sonication before clearance at 35 000 rpm at 4°C for 1 hour. Cleared lysate was loaded on a 5 ml HiTrap TALON Crude column (Cytiva) followed by a wash using 25 ml of H buffer (20 mM HEPES pH 7.5, 5% glycerol, 0.01% NP40, 1 mM β-mercaptoethanol) containing 150 mM NaCl. Top3 protein was eluted with a 50-ml gradient of 0–450 mM imidazole in an H buffer containing 150 mM NaCl. Partially purified protein was further loaded onto a 5 ml HiTrap Heparin HP affinity column (Cytiva) pre-equilibrated in H buffer containing 150 mM NaCl and eluted with an increasing salt gradient to 1 M NaCl. The fractions containing Top3 protein were then loaded onto a 6 ml ResourceS column (Cytiva) pre-equilibrated in an H buffer containing 150 mM NaCl. and eluted with increasing salt gradient to 1 M NaCl. The peak fractions were concentrated on a 30 kDa MWCO Amicon concentrator and applied onto a Superose 6 10/300 column (Cytiva) pre-equilibrated in SEC buffer (20 mM HEPES pH 7.5, 300 mM NaCl, 5% glycerol, 1 mM β-mercaptoethanol, 1 mM TCEP). The fractions containing Top3 were concentrated on a 30 kDa MWCO Amicon concentrator and stored at -80°C in small aliquots.

Rmi1 was produced as an N-terminal GST tag in Hi5 insect cells using the same expression conditions as described above. The cell pellet was resuspended in the lysis buffer (50 mM HEPES pH 7.5, 300 mM NaCl, 5% glycerol, 0.01% NP40, 5 mM β-mercaptoethanol, AEBSF). Resuspended cells were lysed by sonication before clearance at 35 000 rpm at 4°C for 1 h. Cleared lysate was loaded on a 5 ml GSTrap column (Cytiva) followed by wash using 25 ml of H buffer (20 mM HEPES pH 7.5, 5% glycerol, 0.01% NP40, 1 mM β-mercaptoethanol) containing 300 mM NaCl. The Rmi1 protein was eluted with 50 ml of H buffer containing 150 mM NaCl and 100 mM glutathione. Partially purified protein was further loaded onto a 6 ml ResourceS column (Cytiva) pre-equilibrated in H buffer containing 100 mM NaCl and eluted with increasing salt gradient to 1 M NaCl. The peak fractions were concentrated on a 30 kDa MWCO Amicon concentrator and applied onto a Superose 6 10/300 column (Cytiva) pre-equilibrated in SEC buffer (20 mM HEPES pH 7.5, 300 mM NaCl, 5% glycerol, 1 mM β-mercaptoethanol, 1 mM TCEP). The fractions containing GST-Rmi1 were concentrated on a 30 kDa MWCO Amicon concentrator and stored at -80°C in small aliquots. To obtain untagged Rmi1, the concentrated elute fractions from ResourceS column were mixed with 3C HRV protease in a molar ratio of 50:1 and incubated overnight at 4°C. Afterward, the cleaved protein was loaded onto a Superdex 200 10/300 column (Cytiva) with its outlet connected to a 5 ml GSTrap column (Cytiva) pre-equilibrated in SEC buffer (20 mM HEPES pH 7.5, 300 mM NaCl, 5% glycerol, 1 mM β-mercaptoethanol, 1 mM TCEP). The fractions containing untagged Rmi1 were concentrated on a 10 kDa MWCO Amicon concentrator and stored at -80°C in small aliquots.

To purify the Top3-Rmi1 complex, untagged Top3 and N-terminal GST-tagged Rmi1 were cloned into the pBIG1a vector and expressed in Hi5 insect cells using the same expression conditions as described above. The cell pellet was resuspended in the lysis buffer (50 mM HEPES pH 7.5, 300 mM NaCl, 5% glycerol, 0.01% NP40, 5 mM β-mercaptoethanol, AEBSF). Resuspended cells were lysed by sonication before clearance at 35 000 rpm at 4°C for 1 h. Cleared lysate was loaded on a 5 ml GSTrap column (Cytiva) followed by wash using 25 ml of H buffer (20 mM HEPES pH 7.5, 5% glycerol, 0.01% NP40, 1 mM β-mercaptoethanol) containing 300 mM NaCl. The Top3–Rmi1 complex was eluted with 50 ml of H buffer containing 100 mM NaCl and 100 mM glutathione. Partially purified protein was further loaded onto a 6 ml ResourceS column (Cytiva) pre-equilibrated in H buffer containing 100 mM NaCl and eluted with an increasing salt gradient to 800 mM NaCl. The peak fractions were concentrated on a 30 kDa MWCO Amicon concentrator and applied onto a Superose 6 10/300 column (Cytiva) pre-equilibrated in SEC buffer (20 mM HEPES pH 7.5, 300 mM NaCl, 5% glycerol, 1 mM β-mercaptoethanol, 1 mM TCEP). The fractions containing the Top3–Rmi1 complex were concentrated on a 30 kDa MWCO Amicon concentrator and stored at -80°C in small aliquots. To obtain untagged Top3–Rmi1 complex, the concentrated eluted fractions from ResourceS column were mixed with 3C HRV protease in a molar ratio of 50:1 and incubated overnight at 4°C. Afterward, the cleaved protein was loaded onto a Superdex 200 10/300 column (Cytiva) with its outlet connected to a 5 ml GSTrap column (Cytiva) pre-equilibrated in SEC buffer (20 mM HEPES pH 7.5, 300 mM NaCl, 5% glycerol, 1 mM β-mercaptoethanol, 1 mM TCEP). The fractions containing untagged Top3–Rmi1 were concentrated on a 10 kDa MWCO Amicon concentrator and stored at -80°C in small aliquots.

Sgs1 containing an N-terminal 6xHis-MBP tag and C-terminal 6xHis tag was produced in Hi5 cells using the similar expression conditions as described above with a minor change in using 1:300 dilution of baculovirus. The cell pellet (17 g) was resuspended in the lysis buffer (50 mM HEPES pH 7.5, 300 mM NaCl, 5% glycerol, 0.01% NP40, 5 mM β-mercaptoethanol, AEBSF, Serva protease inhibitor cocktail and 1 mM PMSF). Resuspended cells were lysed by sonication before clearance at 35 000 rpm at 4°C for 1 h. Cleared lysate was loaded on a 5 ml MBPTrap column (Cytiva) followed by wash using 35 ml of H buffer (20 mM HEPES pH 7.5, 5% glycerol, 0.01% NP40, 1 mM β-mercaptoethanol) containing 300 mM NaCl. The Sgs1 protein was eluted with a 50-ml gradient of 0–20 mM maltose of H buffer containing 150 mM NaCl. To cleave off N-terminal 6xHis-MBP tag, partially purified protein was mixed with 100 μl 3C HRV protease (6 μg/μl) and incubated overnight at 4°C. Afterward, the cleaved protein was loaded onto a 5 ml HiTrap Heparin HP affinity column (Cytiva) pre-equilibrated in H buffer containing 100 mM NaCl and eluted with an increasing salt gradient from 300 mM to 1 M NaCl. The fractions containing Sgs1 protein were concentrated on a 100 kDa MWCO Amicon concentrator and stored at -80°C in small aliquots. Sgs1-K706A helicase-dead mutant was prepared according to the same protocol as wild type Sgs1 protein.

Sgs1(1-107) fragment containing N-terminal 6xHis tag was produced in *Escherichia coli* strain BL21 STAR. Protein expression was induced by 0.5 mM IPTG at 37°C for 3 h in TB media supplemented with ampicillin (100 μg/ml). The cell pellet was resuspended in the lysis buffer (50 mM HEPES pH 7.5, 300 mM NaCl, 5% glycerol, 0.01% NP40, 5 mM β-mercaptoethanol, AEBSF, Serva protease inhibitor cocktail). Resuspended cells were lysed by sonication before clearance at 35 000 rpm at 4°C for 1 h. Cleared lysate was incubated with 800 μl of Ni-NTA agarose (Qiagen) for 1 h at 4°C. The beads were washed with 20 ml of H buffer containing 150 mM NaCl followed by another wash with 20 ml of H buffer containing 150 mM NaCl and 10 mM imidazole. The protein was eluted in steps with 50, 150, 300 and 500 mM imidazole in H buffer containing 150 mM NaCl. Partially purified protein was further loaded onto a 6 ml ResourceQ column (Cytiva) pre-equilibrated in H buffer containing 150 mM NaCl and eluted with increasing salt gradient to 1 M NaCl. The fractions containing Sgs1 fragment were concentrated on a 10 kDa MWCO Amicon concentrator and applied onto a Superdex 200 10/300 column (Cytiva) pre-equilibrated in SEC buffer (20 mM HEPES pH 7.5, 300 mM NaCl, 5% glycerol, 1 mM β-mercaptoethanol, 1 mM TCEP). The fractions containing Sgs1 protein were concentrated on a 10 kDa MWCO Amicon concentrator and stored at -80°C in small aliquots.

Sgs1(1–605) fragment containing N-terminal 6xHis-MBP tag was produced in *E. coli* strain BL21 STAR. Protein expression was induced by 0.2 mM IPTG at 18°C overnight in TB media supplemented with ampicillin (100 μg/ml). The cell pellet was resuspended in the lysis buffer (50 mM HEPES pH 7.5, 300 mM NaCl, 5% glycerol, 0.01% NP40, 5 mM β-mercaptoethanol, AEBSF, Serva protease inhibitor cocktail). Resuspended cells were lysed by sonication before clearance at 35 000 rpm at 4°C for 1 h. Cleared lysate was loaded on a 5 ml MBPtrap column (Cytiva) followed by first wash using 25 ml of H buffer (20 mM HEPES pH 7.5, 5% glycerol, 0.01% NP40, 1 mM β-mercaptoethanol) containing 500 mM NaCl and the second wash using H buffer containing 150 mM NaCl. The protein was eluted with a 50 ml of H buffer containing 150 mM NaCl and 20 mM maltose. Partially purified protein was further loaded onto a 6 ml ResourceQ column (Cytiva) pre-equilibrated in H buffer containing 150 mM NaCl and eluted with increasing salt gradient to 1 M NaCl. The fractions containing Sgs1 fragment were concentrated on a 30 kDa MWCO Pierce concentrator and incubated with 3C HRV protease in a molar ratio of 50:1 overnight at 4°C. Afterwards, the cleaved protein was applied onto a Superose 6 10/300 column (Cytiva) with its outlet connected to a 5 ml GSTrap column (Cytiva) followed by a 5 ml MBPtrap column (Cytiva). All columns were pre-equilibrated in SEC buffer (20 mM HEPES pH 7.5, 300 mM NaCl, 5% glycerol, 1 mM β-mercaptoethanol, 1 mM TCEP). The fractions containing Sgs1 protein were concentrated on a 50 kDa MWCO Amicon concentrator and stored at -80°C in small aliquots.

Dmc1 was purified as described elsewhere(38) with minor modifications. Briefly, the plasmid expressing Dmc1 protein with N-terminus (His)_6_-affinity tag (a kind gift from Lumir Krejci) was introduced into *E. coli* strain Rosetta(DE3)pLysS. Protein expression was induced by 0.5 mM IPTG at 37°C for 3 h in LB media supplemented with ampicillin (100 μg/ml). The cell pellet was resuspended in the lysis buffer (25 mM Tris–HCl pH 7.5, 500 mM NaCl, 10% glycerol, 0.5 mM EDTA, 0.01% NP40, 1 mM DTT, 1 mM MgCl_2_, 1 mM ATP and AEBSF). Resuspended cells were lysed by sonication before clearance at 35 000 rpm at 4°C for 45 min. Cleared lysate was incubated with 400 μl of Talon Resin (TaKaRa) for 1 h at 4°C. The beads were washed with 10 ml of buffer T (25 mM Tris–HCl pH 7.5, 10% glycerol, 0.5 mM EDTA, 0.01% NP40, 1 mM DTT) containing 150 mM NaCl followed by additional washing step with 10 ml of buffer T containing 500 mM NaCl. The protein was eluted in steps with 200 and 500 mM imidazole in buffer T containing 140 mM NaCl, 1 mM MgCl_2_, and 1 mM ATP. Fractions containing Dmc1 protein were applied onto a 5-ml HiTrap Heparin HP affinity column (Cytiva) equilibrated with buffer T containing 140 mM NaCl, and eluted using a 25-ml gradient of 140–1000 mM NaCl in buffer T containing 1 mM MgCl_2_ and 1 mM ATP. The peak fractions were concentrated on a 30 kDa MWCO Amicon concentrator and applied onto a Superose 6 10/300 column (Cytiva) pre-equilibrated in SEC buffer (20 mM HEPES pH 7.5, 300 mM NaCl, 5% glycerol, 1 mM DTT) supplied with 1 mM MgCl_2_ and 1 mM ATP. The fractions containing Dmc1 were concentrated on a 30 kDa MWCO Amicon concentrator and stored at -80°C in small aliquots.

RPA complex was produced in *E. coli* strain C41 by co-expression of pCOLI-Twin-StrepII-Rfa1, pCDF-6xHis-Rfa2 and pRSF-6xHis-Rfa3 plasmids. Protein expression was induced by 0.5 mM IPTG at 25°C for 3 h in TB media supplemented with ampicillin (100 μg/ml), kanamycin (25 μg/ml) and spectinomycin (50 μg/ml). The cell pellet was resuspended in the lysis buffer (50 mM HEPES pH 7.5, 300 mM NaCl, 5% glycerol, 0.01% NP40, 5 mM β-mercaptoethanol, AEBSF). Resuspended cells were lysed by sonication before clearance at 35 000 rpm at 4°C for 40 min. Cleared lysate was loaded on a 5 ml Strep-Tactin XT column (IBA) followed by wash using 25 ml of H buffer (20 mM HEPES pH 7.5, 5% glycerol, 0.01% NP40, 1 mM β-mercaptoethanol) containing 150 mM NaCl. The protein was eluted with a 50 ml of H buffer containing 100 mM NaCl and 50 mM biotin. Partially purified protein was further loaded onto a 5 ml HiTrap Heparin HP affinity column (Cytiva) pre-equilibrated in H buffer containing 150 mM NaCl and eluted with increasing salt gradient to 1 M NaCl. The peak fractions were concentrated on a 10 kDa MWCO Amicon concentrator and applied onto a Superose 6 10/300 column (Cytiva) pre-equilibrated in SEC buffer (20 mM HEPES pH 7.5, 300 mM NaCl, 5% glycerol, 1 mM β-mercaptoethanol, 1 mM TCEP). The fractions containing the RPA complex were concentrated on a 10 kDa MWCO Amicon concentrator and stored at -80°C in small aliquots.

Rad51 protein was expressed as described previously (39). The cells were lysed in cryo mill and the pellet (80 g) was then resuspended in 150 ml of cell breakage buffer containing 50 mM Tris–HCl pH 7.5, 600 mM KCl, 10% sucrose, 2 mM EDTA, 0.01% NP40, 1 mM β-mercaptoethanol, and AEBSF followed by ultracentrifugation at 35 000 rpm at 4°C for 1 h. The supernatant was subjected to ammonium sulfate precipitation at a concentration of 0.21 g/ml and the precipitate was processed by several rounds of centrifugation at 10 000 rpm at 4°C for 30 min. The ammonium sulfate pellet was dissolved in 140 ml of T buffer (25 mM Tris–HCl, 10% glycerol, 0.5 mM EDTA, pH 7.5, 0.01% NP40, 1 mM DTT) containing AEBSF and applied on a 10 ml Q-Sepharose column (Cytiva). Following the wash step using 30 ml of T buffer containing 140 mM NaCl the protein was eluted by increasing salt gradient to 700 mM NaCl. The fractions containing Rad51 protein were applied onto 5 ml HiTrap Heparin HP affinity column (Cytiva) pre-equilibrated in T buffer containing 50 mM NaCl. The protein was eluted by increasing salt gradient to 900 mM NaCl. Partially purified protein was further loaded onto 6 ml Resource Q column (Cytiva) pre-equilibrated in T buffer containing 100 mM NaCl. The elution was done using increasing salt gradient to 500 mM NaCl. The peak fractions were concentrated on a 30 kDa MWCO Amicon concentrator and applied onto a Superose 6 16/600 column (Cytiva) pre-equilibrated in T buffer containing 300 mM NaCl. The fractions containing Rad51 protein were concentrated on a 30 kDa MWCO Vivaspin concentrator and stored at -80°C in small aliquots.

Rad54 was produced as an N-terminal GST tag in Hi5 insect cells using the same expression conditions as described above except for a shorter expression time (48 h). The cell pellet was resuspended in the lysis buffer (50 mM HEPES pH 7.5, 300 mM NaCl, 5% glycerol, 0.01% NP40, 5 mM β-mercaptoethanol, AEBSF). Resuspended cells were lysed by sonication before clearance at 35 000 rpm at 4°C for 1 h. Cleared lysate was loaded on a 5 ml SP-Sepharose column (Cytiva) pre-equilibrated in H buffer containing 150 mM NaCl and eluted with increasing salt gradient to 1 M NaCl. The fractions containing Rad54 were applied on a 5 ml GSTrap column (Cytiva) followed by wash using 25 ml of H buffer containing 150 mM NaCl. The Rad54 protein was eluted with increasing glutathione gradient to 100 mM. Partially purified protein was further concentrated on a 50 kDa MWCO Amicon concentrator and applied onto a Superose 6 10/300 column (Cytiva) pre-equilibrated in SEC buffer (20 mM HEPES pH 7.5, 300 mM NaCl, 5% glycerol, 1 mM β-mercaptoethanol). The fractions containing GST-Rad54 were concentrated on a 100 kDa MWCO Amicon concentrator and stored at -80°C in small aliquots.

### Mass photometry

Mass Photometry was performed in the mass photometry buffer (MP) containing 30 mM HEPES pH 7.8, 150 mM NaCl, 5% glycerol, 1 mM MgCl_2_ and 1 mM TCEP. Protein samples (3 μM) were pre-equilibrated for 1 h in the MP buffer. Measurements were performed using Refeyn One (Refyn Ltd, Oxford, UK) mass photometer. Directly before the measurement, the sample was diluted 1:100 with the MP buffer. Molecular mass was determined in Analysis software provided by the manufacturer using a NativeMark (Invitrogen) based standard curve created under the identical buffer composition.

### DNA substrates

Fluorescently labelled DNA substrates were prepared as described previously(40,41). The individual DNA substrates were prepared by annealing 5′ fluorescently labelled oligonucleotide 1253 (or 1253-T) with the following oligonucleotides: dsDNA (1253, 1253C); 3′overhang (1253, 3′overhang25nt); Y-form (1253-T, 1254); D-loop (1253-T, 315, 320, X12-3SC), HJ (1253-T, 1254, 1255, 1256). The sequences of all oligonucleotides used in this study are listed in Supplementary Table S2.

### Electrophoretic mobility shift assays

The binding reactions (10 μl volume) were carried out in EMSA buffer (25 mM HEPES pH 7.5, 0.1 μg/μl BSA, 60 mM NaCl) containing indicated fluorescently labelled DNA substrate (10 nM). The reactions were started by addition of increasing amounts of Mer3 protein (37.5, 75, 150 and 300 nM) or Mlh1–Mlh2 complex (20, 40, 75 and 150 nM) and incubated for 20 min at 30°C. After the addition of 2 μl of the gel loading buffer (60% glycerol, 10 mM Tris–HCl, pH 7.4, 60 mM EDTA, 0.15% Orange G), the reaction mixtures were resolved in 0.8% agarose gel in 1× TAE buffer (40 mM Tris, 20 mM acetic acid, 1 mM EDTA). The gels were scanned using Amersham Typhoon scanner (Cytiva) and quantified by ImageQuant TL software (Cytiva).

### Strand separation assays

The strand separation assays (10 μl volume) were carried out in SS buffer (25 mM HEPES pH 7.5, 60 mM NaCl, 0.1 μg/μl BSA, 1 mM MgCl2, 1 mM ATP, 10 mM creatine phosphate, 15 μg/ml creatine kinase) containing indicated fluorescently labelled DNA substrate (5 nM). The reactions were started by addition of increasing amounts of Mer3 protein (10, 20, 40 and 80 nM). After the incubation for 30 min at 30°C the reactions were stopped with 0.5 mg/ml proteinase K and 0.1% SDS, and incubated for 5 min at 37°C. The samples were then mixed with 2 μl of the gel loading buffer (60% glycerol, 10 mM Tris–HCl, pH 7.4, 60 mM EDTA) and separated on 10% (w/v) native polyacrylamide gel in 1× TBE buffer at a constant voltage of 110 V for 1 h at 4°C. The gels were scanned using Amersham Typhoon scanner (Cytiva) and quantified by ImageQuant TL software (Cytiva).

### Cross-linking mass spectrometry (XL-MS)

For XL-MS analysis proteins were diluted in 200 μl of XL-MS buffer (30 mM HEPES 6.8, 150 mM NaCl, 5% glycerol, 1 mM MgCl_2_, 1 mM TCEP) to the final concentration of 3 μM, mixed with 3 μl of disuccinimidyl dibutyric urea (DSBU) (200 mM) and incubated for 1 h at 25°C. The reaction was stopped by adding 20 μl of 1 M Tris–HCl pH 8.0 and incubated for another 30 min at 25°C. The crosslinked sample was precipitated by addition of 4× volumes of 100% cold acetone followed by overnight incubation at -20°C. Samples were analysed as previously described (42), making use of MeroX (43) for data analysis, which only assumes that one of the two cross-linked residues must be a primary amine. Each sample was measured twice in independent cross-linking experiments. For interaction network visualisation XVis software was used and for visualisation of the crosslinks on the PDB model PyXlinkViewer (44) and XMAS (45) was used. Each time a different cutoff for the cross-linking credibility was selected depending on the quality of the cross-linking data. In all figures only cross-links that were measured in two independent datasets are shown.

### SEC-MALS

50 μl samples at 10 μM concentration were loaded onto a Superose 6 5/150 (for the full length protein) analytical size exclusion column (Cytiva) equilibrated in SEC-MALS buffer (30 mM HEPES pH 6.8, 300 mM NaCl, 1 mM MgCl2, 5 μM ZnCl_2_, 1 mM TCEP) attached to a 1260 Infinity II LC System (Agilent). MALS was carried out using a Wyatt DAWN detector attached in line with the size exclusion column. Mer3 fragments were analysed on Superdex 200 5/150 column (Cytiva) equilibrated in SEC-MALS2 buffer (50 mM HEPES pH 7.5, 300 mM NaCl, 1 mM TCEP).

### Alphafold2 predictions

The predicted structure of full-length Mer3 was obtained from the publicly available database (https://www.alphafold.ebi.ac.uk/ ID P51979). The predicted structures of Mlh1/Mlh2, Top3/Rmi1 and Sgs1/Top3/Rmi1 were calculated using AlphaFold Multimer (2.2.0)(46) run on GPU nodes of the Raven HPC of the Max Planck Computing and Data Facility (MPCDF), Garching. Each job was run on a single node consisting of 4× Nvidia A100 NVlink 40GB GPUs. Multiple predictions were generated for each run, and the best model (determined by pTM score) was then used. PAE plots were generated using a custom script (Vikram Alva, MPI Biology Tübingen).

### Pull-down assays

For pull-down between Mer3 and Top3–Rmi1, GST-tagged Top3–Rmi1 (0.8 μM) was incubated with Strep-tagged Mer3 (0.7 μM) in the reaction buffer (25 mM HEPES pH 7.5, 5% glycerol, 100 mM NaCl, 1 mM TCEP, 0.1% Tween-20) for 20 min at 30°C in the thermomixer (950 rpm). Pre-washed magnetic glutathione beads (1 μl) were then added to the samples and the mixtures were incubated for 2 min at 30°C in the thermomixer (750 rpm). Beads were washed twice with 100 μl of the reaction buffer. The proteins were eluted by boiling in 30 μl 2× SDS Laemmli buffer. The samples were loaded onto 10% SDS-PAGE and stained by Der Blaue Jonas gel dye.

For pull-down between Top3 and Mer3, His-tagged Top3 (1.3 μM) was incubated with Strep-tagged Mer3 (0.7 μM) in the reaction buffer (25 mM HEPES pH 7.5, 5% glycerol, 50 mM NaCl, 1 mM TCEP, 0.1% Tween-20) for 20 min at 30°C in the thermomixer (950 rpm). Anti-Strep antibody (2 μl, Abcam, ab76949) was added to the reactions and the mixtures were incubated for 1 h at 4°C in the thermomixer (950 rpm). Finally, 0.5 μl pre-washed magnetic-conjugated protein G beads (Dynabeads protein G, Invitrogen) were added to the reactions followed by the incubation for 1 hour at 4°C in the thermomixer (950 rpm). Beads were washed twice with 100 μl of the reaction buffer. The proteins were eluted by boiling in 30 μl 2× SDS Laemmli buffer. The samples were loaded onto 11% SDS-PAGE and analysed by western blot. After semi-dry western blotting, proteins were incubated with primary antibodies Strep-Tag II (1:1000; Merck, 71590) or anti-His (1:1000, Qiagen, 34660), respectively. Following the incubation with the secondary HRP-conjugated anti-mouse antibodies (1:7500; Merck, 401215), the proteins were visualized by chemiluminescence detection (ECL Prime Western Blotting detection reagent, Cytiva).

For pull-down between Rmi1 and Mer3, GST-tagged Rmi1 (2.6 μM) was incubated with Strep-tagged Mer3 (1 μM) in the reaction buffer (25 mM HEPES pH 7.5, 5% glycerol, 75 mM NaCl, 1 mM TCEP, 0.1% Tween-20) for 20 min at 30°C in the thermomixer (950 rpm). Pre-washed glutathione beads (10 μl) were then added to the samples and the mixtures were incubated for 30 min at 7°C in the thermomixer (950 rpm). Beads were washed twice with 100 μl of the reaction buffer. The proteins were eluted by boiling in 30 μl 2× SDS Laemmli buffer. The samples were loaded onto 11% SDS-PAGE and stained with coomassie brilliant blue.

For pull-down between Mer3 and Top3–Rmi1 in the presence or absence of Sgs1(1–107), GST-tagged Top3–Rmi1 (1 μM) was incubated with Strep-tagged Mer3 (1 μM) in the reaction buffer (25 mM HEPES pH 7.5, 5% glycerol, 100 mM NaCl, 1 mM TCEP, 0.1% Tween-20) for 20 min at 30°C in the thermomixer (950 rpm). For competition assays, increasing amounts of His-tagged Sgs1(1–107) (8 or 25 μM) were added to the reactions. Pre-washed glutathione beads (10 μl) were then added to the samples and the mixtures were incubated for 30 min at 7°C in the thermomixer (950 rpm). Beads were washed twice with 100 μl of the reaction buffer. The proteins were eluted by boiling in 30 μl 2× SDS Laemmli buffer. The samples were loaded onto 11% or 13% SDS-PAGE and stained with coomassie brilliant blue.

For pull-down between de-/phosphorylated variants of Mer3 and Top3–Rmi1 in the presence or absence of Sgs1(1–605), GST-tagged Top3–Rmi1 (0.8 μM) was incubated with Strep-tagged Mer3 (0.8 μM) in the reaction buffer (25 mM HEPES pH 7.5, 5% glycerol, 100 mM NaCl, 1 mM TCEP, 0.1% Tween-20) for 20 min at 30°C in the thermomixer (950 rpm). For competition assays, increasing amounts of untagged Sgs1(1–605) (1.5 or 6 μM) were added to the reactions. Pre-washed magnetic glutathione beads (1 μl) were then added to the samples and the mixtures were incubated for 2 min at 30°C in the thermomixer (750 rpm). Beads were washed twice with 100 μl of the reaction buffer. The proteins were eluted by boiling in 30 μl 2× SDS Laemmli buffer. The samples were loaded onto 10% SDS-PAGE and stained by Der Blaue Jonas gel dye.

For pull-down between Mlh1-Mlh2 and Mer3, MBP-tagged Mlh1-Mlh2 complex (0.1 μM) was incubated with Strep-tagged Mer3 (0.3 μM) in the reaction buffer (25 mM HEPES pH 7.5, 100 mM NaCl, 1 mM MgCl_2_, 1 mM DTT) for 20 min at 30°C in the thermomixer (950 rpm). Anti-MBP antibody (0.5 μl, Invitrogen, PA1-989) was added to the reactions and the mixtures were incubated for 1 h at 4°C in the thermomixer (750 rpm). Finally, 1 μl pre-washed magnetic-conjugated protein G beads (Dynabeads protein G, Invitrogen) were added to the reactions followed by the incubation for 1 h at 4°C in the thermomixer (750 rpm). Beads were washed twice with 100 μl of the reaction buffer. The proteins were eluted by boiling in 30 μl 2× SDS Laemmli buffer. The samples were loaded onto 9% SDS-PAGE and analysed by western blot. After semi-dry western blotting, proteins were incubated with primary antibodies Strep-Tag II (1:1000; Merck, 71590) or anti-MBP (1:1000, New England Biolabs, E8032), respectively. Following the incubation with the secondary HRP-conjugated anti-mouse antibodies (1:7500; Merck, 401215), the proteins were visualized by chemiluminescence detection (ECL Prime Western Blotting detection reagent, Cytiva). Pull-down between Mer3 (0.3 μM), Mlh1–Mlh2 (0.1 μM) and Top3/GST-Rmi1 complex (0.3 μM) was done using the same protocol but in reaction buffer containing 25 mM HEPES pH 7.5, 5% glycerol, 75 mM NaCl, 1 mM TCEP, 0.1% Tween-20. For detection of GST-Rmi1, proteins were incubated with anti-GST primary antibody (1:1000; Merck, G7781) followed by the incubation with the secondary HRP-conjugated anti-rabbit antibodies (1:7500; Merck, 401353). For competition pull-down GST-tagged Top3-Rmi1 (0.8 μM) was incubated with Strep-tagged Mer3 (0.2 or 0.8 μM) in the presence or absence of Mlh1–Mlh2 (0.8 μM) in the reaction buffer (25 mM HEPES pH 7.5, 5% glycerol, 100 mM NaCl, 1 mM TCEP, 0.1% Tween-20) for 20 min at 30°C in the thermomixer (950 rpm). Pre-washed glutathione magnetic beads (1 μl) were then added to the samples and the mixtures were incubated for 1.5 min at 30°C in the thermomixer (750 rpm). Beads were washed twice with 100 μl of the reaction buffer. The proteins were eluted by boiling in 30 μl 2× SDS Laemmli buffer. The samples were loaded onto 10% SDS-PAGE and stained with Der Blaue Jonas (Coomassie Brilliant Blue) gel dye.

For the pull-down between Mer3 and Dmc1 or Rad51, His-tagged Dmc1 (3.2 μM) or untagged Rad51 (2.8 μM) were incubated with Strep-tagged Mer3 (0.7 μM) in the reaction buffer (25 mM HEPES pH 7.5, 5% glycerol, 100 mM NaCl, 1 mM TCEP, 0.1% Tween-20) for 20 min at 30°C in the thermomixer (750 rpm). Finally, 1 μl pre-washed Pierce Streptavidin magnetic beads (Thermo Fisher Scientific) were added to the reactions and the mixtures were incubated for 1.5 min at 4°C in the thermomixer (950 rpm). Beads were washed twice with 100 μl of the reaction buffer. The proteins were eluted by boiling in 30 μl 2× SDS Laemmli buffer. The samples were loaded onto 10% SDS-PAGE and stained by Der Blaue Jonas (Coomassie Brilliant Blue) gel dye.

### Microscale thermophoresis (MST)

Binding affinity analysis by microscale thermophoresis was performed using the Monolith NT instrument (Nanotemper Technologies). All reactions (in triplicates) were done in the commercial MST buffer (50 mM Tris–HCl pH 7.4, 150 mM NaCl, 10 mM MgCl_2_; Nanotemper Technologies) supplied with 0.05% Tween-20. Measurements were performed at 25°C, and contained constant concentration of 45 nM RED-NHS labelled Mer3 (labelling was performed according to the manufacturer's protocol – Nanotemper Technologies) and increasing concentrations of Top3/Rmi1, Rmi1 or Mlh1/Mlh2, respectively. To analyze the binding affinity between Sgs1 and Top3–Rmi1, the measurements were performed using 30 nM RED-NHS labelled Sgs1(1–605) and increasing concentrations of Top3–Rmi1. Data were analysed by the MO.Affinity Analysis software (NanoTemper Technologies).

### D-loop assay

The reactions (in a total volume 11 μl) were performed in D-loop reaction buffer (25 mM HEPES pH 7.5, 0.1 μg/μl BSA, 100 mM NaCl, 1 mM MgCl_2_, 0.1 mM CaCl_2_, 1 mM ATP, 10 mM creatine phosphate, 15 μg/ml creatine kinase). Radioactively labelled 90-mer ssDNA (oWL981, 2 μM nucleotides) was incubated with Dmc1 protein (1.5 μM) for 5 min at 37°C followed by addition of RPA (90 nM) and additional incubation for 5 min at 37°C. The formation of D-loop was started by addition of pUC19 plasmid (18 nM molecules). After 15 min incubation at 30°C, Sgs1 (15 nM), Top3–Rmi1 (15 nM) and the increasing amounts of Mer3 and/or Mlh1/Mlh2 (15, 150, 300 nM) were added to the reactions. At the indicated time points, 10.5 μl of the sample was mixed with 0.1% SDS (final) and 0.5 mg/ml proteinase K followed by incubation for 15 min at 37°C. The deproteinized samples were separated in a 0.9% agarose gel. After electrophoresis, the gel was dried on grade 3 chromosome paper (Whatman), exposed to a phosphorimager screen, and visualised using Amersham Typhoon scanner (Cytiva). The quantification was done using ImageQuant TL software (Cytiva).

For Rad51-mediated D-loop assays, the reactions (in a total volume 11 μl) were performed in the reaction buffer containing 25 mM HEPES pH 7.5, 0.1 μg/μl BSA, 100 mM NaCl, 1 mM MgCl_2_, 1 mM ATP, 10 mM creatine phosphate, 15 μg/ml creatine kinase). Radioactively labelled 90-mer ssDNA (oWL981, 2 μM nucleotides) was incubated with Rad51 protein (1.5 μM) for 5 min at 37°C followed by addition of RPA (90 nM) for 5 min at 37°C and Rad54 (400 nM) for additional incubation for 3 min at RT. The formation of D-loop was started by addition of pUC19 plasmid (18 nM molecules). After 15 min incubation at 30°C, Sgs1 (15 nM), Top3–Rmi1 (15 nM) and the increasing amounts of Mer3 (15, 150, 300 nM) were added to the reactions. At the indicated time points, 10.5 μl of the sample was mixed with 0.2% SDS (final) and 1 mg/ml proteinase K followed by incubation for 15 min at 37°C. The samples were analyzed as described above.

### Yeast strains

All strains, except those used for Y2H analysis and Rad51 expression, were derived from *S. cerevisiae* SK1 strains YML4068 and YML4069 (a kind gift from Joao Matos) and their genotypes are listed in Supplementary Table S3. C-terminal tagging of Mer3 and Top3 was done using the PCR-based method as previously described(47,48). Mer3 was tagged with 9 copies of Myc tag by using plasmid pYM18. Top3 was tagged by 3 copies of HA tag using the plasmid pYM24. The correct epitope tag insertion was confirmed by PCR.

### Yeast two-hybrid

Yeast genes ORFs were PCR-amplified from SK1 strain genomic DNA. MER3 was prepared by Gibson assembly of 2 PCR products eliminating MER3′s intron. The corresponding genes were cloned into pGAD-C1 or pGBDU-C1 vectors, respectively. The resulting plasmids were co-transformed into the *S. cerevisiae* reporter strain (yWL365; a kind gift from Gerben Vader) and plated onto the selective medium lacking leucine and uracil. For drop assay, 2.5 μl from 10-fold serial dilutions of cell cultures with the initial optical density (OD_600_) of 0.5 were spotted onto -Leu/-Ura (control) and -Leu/-Ura/-His plates with or without 1 mM 3-aminotriazole. Cells were grown at 30°C for up to 4–6 days. and imaged.

### Meiotic time course

Cells were grown overnight in liquid YPD culture at 30°C followed by inoculation in pre-sporulation media (BYTA; 50 mM potassium phthalate, 1% yeast extract, 2% bacto tryptone, and 1% potassium acetate) at OD_600_ = 0.3 for additional 16–18 h at 30°C. Next morning, cells were washed twice with sporulation medium (SPO, 0.3% potassium acetate) and resuspended in sporulation medium at OD_600_ = 1.9 to induce meiosis at 30°C.

### *In vivo* co-immunoprecipitation

100 ml of meiotic cultures (at 6 h into a meiotic time course) were harvested by spinning down at 3000 rpm for 5 min followed by washing with 500 μl of cold H_2_O containing 1 mM PMSF. Cell pellets were resuspended in 350 μl of ice-cold co-IP buffer (50 mM Tris–HCl pH 7.5, 150 mM NaCl, 1% Nonidet *P*-40, 1 mM EDTA pH 8.0, 1 mM PMSF, AEBSF, Serva protease cocktail and a cocktail of protease inhibitors which was freshly added) and glass beads. The cells were lysed using a FastPrep-24 disruptor (MP Biomedicals) (setting: 2 × 40 s cycles at speed 6.0). Lysates were cleared by two rounds of centrifugation for 10 min at 15 000 rpm and the supernatants were after each centrifugation step transferred to a clean microcentrifuge tube. 1 μl of antibody (anti-HA; Sigma-Aldrich H6908) was added to the samples followed by 3 h incubation at 4°C. Subsequently, 25 μl of buffer-washed Dynabeads Protein G (Thermo Fisher Scientific) was added and the samples were incubated overnight at 4°C. The next day, Dynabeads were washed four times with 500 μl of ice-cold IP buffer. For the final wash, beads were transferred to a new microcentrifuge tube and washed 500 μl of ice-cold IP buffer without Nonidet *P*-40. The beads were resuspended in 55 μl of 2× SDS Laemmli buffer and incubated for 5 min at 95°C. The samples were loaded onto a 9% SDS-polyacrylamide gel and blotted to nitrocellulose membrane. Antibodies used were as follows: anti-PGK1 (22C5D8, Thermo Fisher Scientific, 459250, 1:1000), anti-HA (Sigma-Aldrich, H6908, 1:1000), anti-Myc (Abcam, ab1326, 1:1000), goat anti-rabbit IgG peroxidase conjugate (Merck, 401353), goat anti-mouse IgG peroxidase conjugate (Merck, 401215). Signal was detected using ECL Prime Western Blotting Detection Reagents (Cytiva) and visualised by a ChemiDocMP (Bio-Rad Inc).

### Phosphorylation analysis of purified Mer3 and yeast lysates by LC-MS/MS

Independent purifications of Mer3 from insect cells without treatment, treated with ocadaic acid or lambda phosphatase (*n* = 3 from each condition) were analyzed by LC-MS/MS to identify phosphorylation sites. Samples were denatured in 8M Urea and then reduced, alkylated and digested with LysC/Trypsin. Obtained peptides were concentrated and desalted on C18 StageTips.

For the enrichment of phosphorylated peptides from yeast whole lysates, yeast cells (harvested after 6 h in sporulation medium; yWL429) were lysed in 2% SDC containing 100 mM Tris pH 8.5, sonicated in a bioruptor (10 min; duty cycle: 30 s on/30 s off) and boiled at 95°C for 5 min. 500 μg of yeast lysate per replicate (*n* = 3) were enriched for phosphopeptides using the EasyPhos method as described (49).

100 ng of obtained peptides from purified Mer3 were separated on an UltiMateTM 3000 HPLC System (ThermoFisher Scientific) using a 45 min gradient from 5–60% acetonitrile with 0.1% formic acid. Phosphopeptide enrichments from yeast lysates were separated on an EASY-nLC 1200 HPLC system (ThermoFisher Scientific) using a 120 min gradient from 5 to 40% acetonitrile with 0.1% formic acid. Peptides were directly sprayed via a nano-electrospray source in an Q Exactive HF-X Spectrometer (ThermoFisher Scientific) (for purified Mer3) or in an Orbitrap ExplorisTM 480 (ThermoFisher Scientific) (for phosphopeptide enrichments from lysates). Data were acquired in a data-dependent mode acquiring one survey scan (MS scan) and subsequently 15 MS/MS scans of the most abundant precursor ions from the survey scan. The mass range was set to *m*/*z* 300 to 1600 and the target value to 3 × 106 precursor ions with 1.4 Th isolation window. The maximum injection time for purified samples was 28 msec and for phosphopeptides from lysates 120 ms for MS and MS/MS scans. MS scans were recorded with a resolution of 60 000 and MS/MS scans with 15 000. Unassigned precursor ion charge states and singly charged ions were excluded. To avoid repeated sequencing, previously sequenced ions were dynamically excluded for 30 s.

Resulting raw files were processed with the MaxQuant software (version 1.6.14) using a reduced database containing only the proteins present in the samples (purified Mer3) and for the enriched phosphopeptides from yeast lysates a database for *S. cerevisiae* SK1 for the search(50,51). Oxidation (M) and phosphorylation (STY) were given as variable modifications and carbamidomethylation (C) as fixed modification. A false discovery rate cut-off of 1% was applied at the peptide and protein levels and as well on the phosphorylation peptides.

The MaxQuant Phospho (STY)sites.txt output table was then further processed in Peresus (version 1.6.50) (52). Contaminants and reverse hits were removed. To obtain a list of high-confident phosphorylation sites of purified Mer3, phosphopeptides were filtered for a localization probability of >99% and the phosphopeptide had to be present in purified Mer3 (at least in two replicates out of three) and to show at least a 2-fold increase with okadaic acid treatment. Phosphopeptides from the *in vivo* phospho enrichment from lysates were filtered for at least two identifications out of three replicates.

## RESULTS

### Hybrid structural and biophysical analysis of Mer3

We set out by purifying full-length *S. cerevisiae* Mer3 from baculovirus-infected insect cells using a COOH-terminal 2xStrep-II tag. Using a 3-step purification (see materials and methods) we were able to produce a Mer3 that was homogenous and devoid of nucleic acid contamination (Figure 1B). Using mass photometry, we determined our protein preparation to be a homogenous sample of monomers of Mer3 in the solution at a concentration of ∼30 nM (Figure 1C). We tested the DNA binding of recombinant Mer3 and found that it bound both single-stranded DNA (ssDNA) and synthetic ‘D-loop’ substrates with high affinity (Supplementary Figure S1A). Tight binding to D-loops is consistent with previous studies (31,32), but high affinity binding to ssDNA was not reported to date. We also confirmed that our recombinant Mer3 preparation was catalytically active in a synthetic D-loop unwinding assay (Supplementary Figure S1B).

To probe the structure of Mer3, we made use of the AlphaFold2 (AF2) (53) predicted model that is publicly available (AlphaFold EBI ID P51979). Based on the pLDDT score and the predicted error alignment (PAE) plots the AF2 model of Mer3 is of an overall high quality (Supplementary Figure S1C and D). The predicted model reveals an architecture with mostly unstructured N- and C-terminal regions and a large structured core consisting of (from N- to C-terminus) RecA-like 1, RecA-like 2, winged helix (WH), helical bundle (HB), helix-loop-helix (HLH) and Ig-like domains (Figure 1D). A DALI (54) search reveals the greatest similarity to the spliceosomal helicase Brr2 (55), in particular the presence of the HB, HLH and Ig-like domains in what has been previously described as a Sec63 like-region (55,56).

We tested the AF2 model prediction using chemical cross-linking coupled to mass-spectrometry (XL-MS). We used DSBU (disuccinimidyl dibutyric urea) (42,57) as a bifunctional chemical cross-linker to produce an inter-residue distance map of Mer3 (Supplementary Figure S1E). While DSBU preferentially cross-links primary amines (Lys and amino termini), we also observed cross-links between primary amines and free hydroxyl group side chains (Thr, Ser, Tyr), as described previously (42). We modelled the cross-links onto the AF2 model of Mer3, and determined which cross-links were consistent with the inter-residue distances in the AF2 model, and which were violated. Surprisingly, despite the high quality of the AF2 prediction, a proportion of Mer3 observed cross-link distances were not compatible with the model (distances of >30 Å, Figure 1E and 1F). Closer analysis of the cross-linking data revealed a number of ‘self’ cross-linked peptides that could only be compatible with a higher order stoichiometry (Figure 1F). This is superficially surprising, since we had already demonstrated that Mer3 is a monomer (Figure 1C), however at a ∼100-fold lower concentration than we used for XL-MS (30 nM in the mass photometer versus 3 μM in the reaction with DSBU). Likewise the elution volume of Mer3 in preparative size exclusion chromatography is smaller than the 158 kDa marker and would not be inconsistent with a dimer of Mer3 (Figure 1B). Since a sample concentration of 3 μM is not currently compatible with the mass-photometry method, we instead used multi-angle light scattering coupled to size-exclusion chromatography (SEC-MALS) to measure the molecular mass of Mer3 at higher (10 μM at injection) concentrations. SEC-MALS revealed a single species with a molecular mass of 266 kDa, consistent with a dimer of Mer3 (theoretical molecular mass 277.6 kDa) (Figure 1G). As such we conclude that Mer3 forms dimers at higher concentrations with a *K*_D_ in the high nM to low μM range. A meta analysis of protein abundance datasets suggested that there are ∼1540 molecules of Mer3 in a diploid meiotic cell, which, if all Mer3 is localised to the nucleoplasm, would give a concentration of ∼800 nM (58), and therefore not inconsistent with the existence of Mer3 dimers *in vivo*.

To map the possible oligomerization region we analysed Mer3 truncations. Constructs of Mer3 that disrupted the structural core of Mer3 were unstable in recombinant preparations. Instead we made use of constructs lacking the N- or C-terminal unstructured regions: Mer3ΔN (122–1187), Mer3ΔC (1–1023), based on the AF2 prediction. We carried out initial tests using yeast-two-hybrid (Figure 1H and Supplementary Figure S2) which suggested that both the N- and C-terminal unstructured regions contributed to the self association of Mer3. We purified truncations of Mer3 lacking the N-terminus (Mer3ΔN), the C-terminus (Mer3ΔC) and found that either of these truncations perturbed Mer3 dimer formation as determined by SEC-MALS (Figure 1G), though also not producing monomeric Mer3. A double truncation (Mer3ΔNΔC), did also not run as a true monomer (Figure 1I). As such we conclude that Mer3 forms dimers at higher concentrations, and that this dimerisation requires both the N- and C-terminal unstructured regions, possibly indicating at least in part a *trans* N- to C-terminal interaction.

### Biophysical and structural analysis of Mer3 interaction with Mlh1 and Mlh2

**Figure 2.**
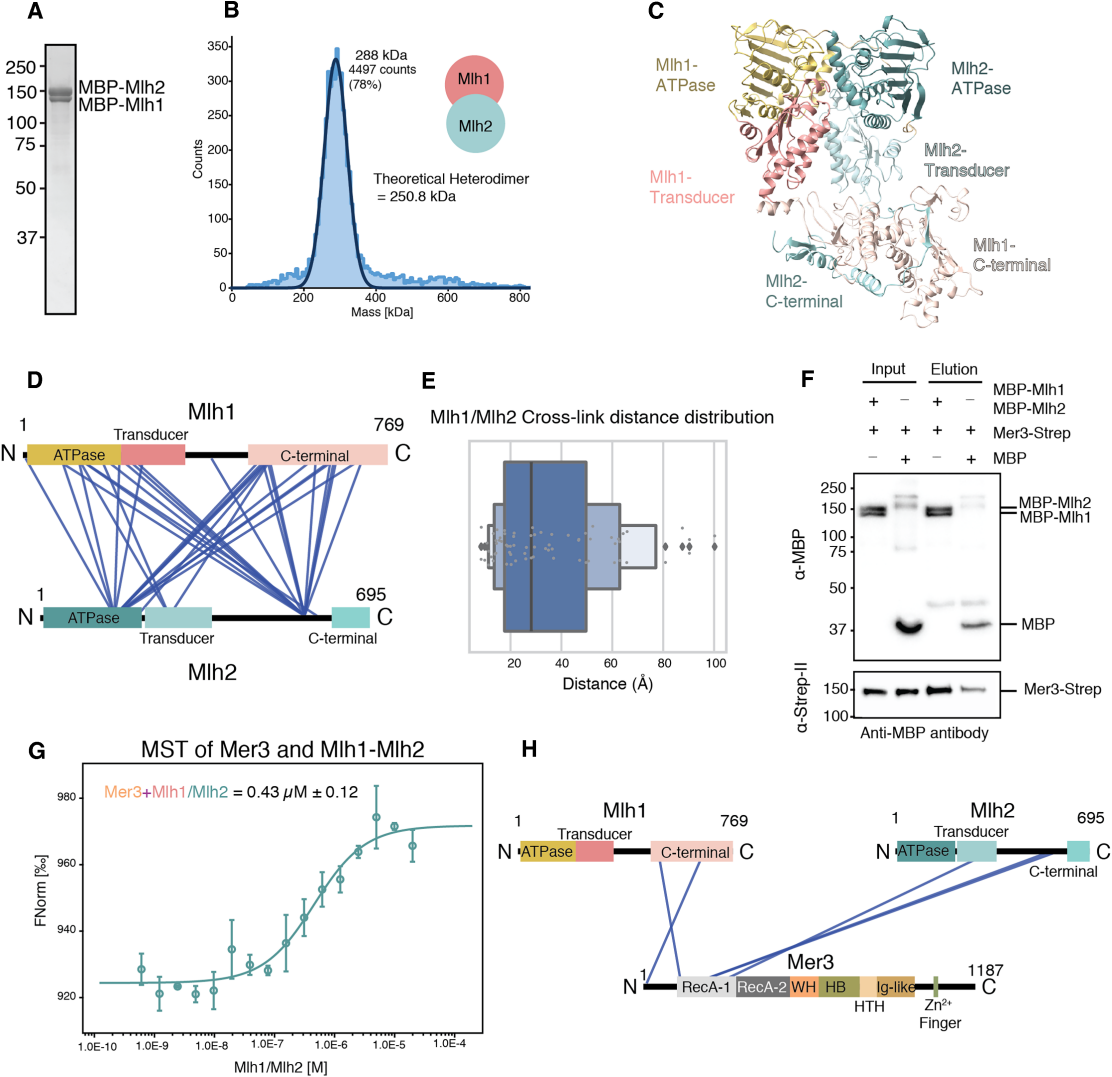
Analysis and quantification of the Mer3/Mlh1/Mlh2 complex. (**A**) Coomassie stained SDS-PAGE showing a representative purification of MBP-Mlh1/MBP-Mlh2 complex (See Supplementary Figure S3A for corresponding chromatograph). (**B**) Mass Photometry profile of purified MBP-Mlh1/MBP-Mlh2 complex (30 nM concentration). We recorded a single species with a molecular mass consistent with a heterodimer of Mlh1/Mlh2. (**C**) AlphaFold2 Multimer(46) model of *S. cerevisiae* Mlh1/Mlh2 heterodimer (see Supplementary Figure S3B and C for quality assessment). Mlh1 is coloured in mustard and pink, and Mlh2 in cyan. Both proteins share the same domain organisation, from N- to C-terminal; ATPase domain, Transducer domain, C-terminal domain. (**D**) Visualisation of XL-MS data from purified MBP-Mlh1/MBP-Mlh2 complex, created using XVis (71). Domains are coloured as in C). MBP is removed from the visualisation due to the inability to unambiguously assign cross-links. Intra-chain cross-links are not shown for greater clarity. Only high confidence cross-links (score > 50; FDR < 1%) common to two independent datasets are shown. (**E**) Cross-link distance distribution profile from XMAS (45), based on the AF2 model shown in C) using cross-links common between two independent replicated datasets. Here we indicate that the majority of cross-links are within the accepted distance for the DSBU cross-linker. The exceptions are most likely due to the flexibility of the C-terminal domains of Mh1 and Mlh2 relative to the N-terminus. (**F**) Pulldown assay (binding to anti-MBP), visualised with western blotting (anti-MBP against MBP-Mlh1 and MBP-Mlh2; anti-Strep against Mer3-Strep), to confirm that our recombinant MBP-Mlh1/MBP-Mlh2 complex (0.1 μM) binds to Mer3 (0.3 μM) as previously shown(31). (**G**) Microscale thermophoresis (MST) analysis of Mer3 binding to MBP-Mlh1/MBP-Mlh2 complex. Mer3 was fluorescently labelled (see methods) and MBP-Mlh1/MBP-Mlh2 titrated against Mer3. Experiments were carried out in triplicate and the *K_D_* of 0.43 μM was determined from the fitting curve in the NanoTemper Affinity Analysis v2.3 software (NanoTemper Technologies GmbH). (**H**) Visualisation of XL-MS data of the DSBU cross-linked Mer3/MBP-Mlh1/MBP-Mlh2 complex. MBP is removed from the visualisation due to the inability to unambiguously assign cross-links. Intra-chain cross-links are not shown for greater clarity. Only high confidence cross-links (score > 50; FDR < 1%) common to two independent datasets are shown.

It was previously shown that Mer3 binds directly to the Mlh1/Mlh2 complex and that this interaction plays a role in regulating the size of gene conversions for both COs and NCOs (31). To investigate the structural organisation of this complex we purified a complex of MBP-Mlh1 and MBP-Mlh2 from insect cells, again making use of a 3-step purification to ensure that it was free of nucleic acids (Figure 2A, Supplementary figure S3A). Both proteins were 6xHis-MBP tagged because purification of these proteins without the solubilization tag resulted in a much lower yield and in our experiments, we also left the MBP-tag on both proteins for the downstream experiments due to continued problems with solubility. We were also able to produce His/Strep-tagged versions of Mlh1/Mlh2, and compared the DNA binding of the two differently tagged versions of Mlh1/Mlh2 complex (Supplementary Figure S3B), which showed robust binding to D-loops, but more limited binding to dsDNA and ssDNA, regardless of the tag used. Using mass photometry we determined the molecular mass of the purified MBP-Mlh1/MBP-Mlh2 complex to be 289 kDa; consistent with a heterodimer (theoretical mass of a heterodimer = 250.8 kDa) (Figure 2B). While adding fusion tags to the termini of Mlh1 results in a loss of function *in vivo*, necessitating an internal-tagging strategy recombinant N-terminally tagged proteins have at least recapitulated the interaction with Mer3 helicase (31). Nonetheless the presence of N-terminal tags on Mlh1/Mlh2 complex may interfere with additional protein-protein interactions, whereas C-terminal tags on Mlh1 might interfere with its role in other complexes, for example with Pms1(59).

The publicly available AlphaFold2 models are currently based on monomeric proteins. To better interpret the results from XL-MS we made an AF2 model of Mlh1/Mlh2 using AlphaFold2 multimer(46) (Figure 2C). The multimeric model prediction quality was high for the ATPase and transducer regions of both Mlh1 and Mlh2 (predicted aligned error < 10 Å, pLDDT > 50) (Supplementary Figure S3C and D). Both Mlh1 and Mlh2 have the same domain organisation; an N-terminal ATPase domain, followed by a transducer domain, an unstructured region, and a C-terminal domain (CTD). The structure of the C-term domain of Mlh1 protein was predicted to be accurate however the general orientation, relative to the ATPase domain and the transducer domain, was low of confidence based on the PAE plot. The C-term domain of Mlh2, as well as the unstructured regions of both proteins, couldn’t be predicted with high confidence.

We characterised the Mlh1/Mlh2 complex using XL-MS and the DSBU crosslinker (Figure 2D). We observed that the majority of the high confidence crosslinks detected between Mlh1 and Mlh2 are broadly distributed on the sequence of the Mlh1 protein whereas they concentrate on two distinctive locations in Mlh2. One of them is in the ATPase domain (K159) and the other one is the N-terminal region of the C-terminal domain (K560) indicating that these two regions of the Mlh2 may be involved in interaction with Mlh1. The N-terminal ATPase-transducer domains of Mlh1 and Mlh2 form a heterodimer that is nearly identical to the N-terminal domain of homodimeric MutL protein (RMSD 1.07Å over 220 amino acids) (60). We plotted intra- and inter-chain crosslinks on the AF2 Mlh1/Mlh2 model, and found that the overall distribution of cross-link distances is consistent with a generally accurate model (Figure 2E). However a number of long-distance outliers point towards some flexibility within the structure, particularly for the unstructured regions and the C-terminal domain.

In order to further characterise Mer3 and Mlh1/Mlh2 interaction, we first performed an *in vitro* pull-down assay to confirm that our purified proteins can form a complex (Figure 2F). We characterised the strength of this interaction using microscale thermophoresis (MST) utilising fluorescently labelled Mer3, and determined a *K*_D_ of 436 ± 122 nM (Figure 2G). Next, we determined the structural organisation of the reconstituted Mer3/Mlh1/Mlh2 heterotrimeric complex using XL-MS (Figure 2H). We observed relatively few cross-links that were common between two datasets, but the highest-confidence cross-links were between Mlh2 and the RecA-1 domain of Mer3 and Mlh1 and the N-terminally unstructured region (Figure 2H). It was previously shown that Mer3 interacted with the Mlh1/Mlh2 complex, at least in part via a conserved region of the Mer3 Ig-like domain (31). To examine this we also pooled the cross-links from the two independent cross-linking mass-spec experiments, which provided far more cross-links between Mer3 and Mlh1/Mlh2 (Supplementary Figure S3E). With this approach we observed several cross-links between the Mer3 Ig-like domain and both Mlh1 and Mlh2. We mapped the Mlh1/Mlh2 cross-links onto the surface of the Mer3 AF2 model (Supplementary Figure S3F). The surface mapping showed cross-linked residues clustering on the RecA-1, WH, HTH and Ig-like domains. Previous work showed that Mer3 R893, which when mutated to glutamic acid, ablates the interaction between Mer3 and Mlh1/Mlh2 (31). Consistent with this observation R893 lies at the centre of cross-linked residues of Mer3 (Supplementary Figure S3F).

### Mer3 interacts with Top3, Rmi1 and the Top3-Rmi1 complex

**Figure 3.**
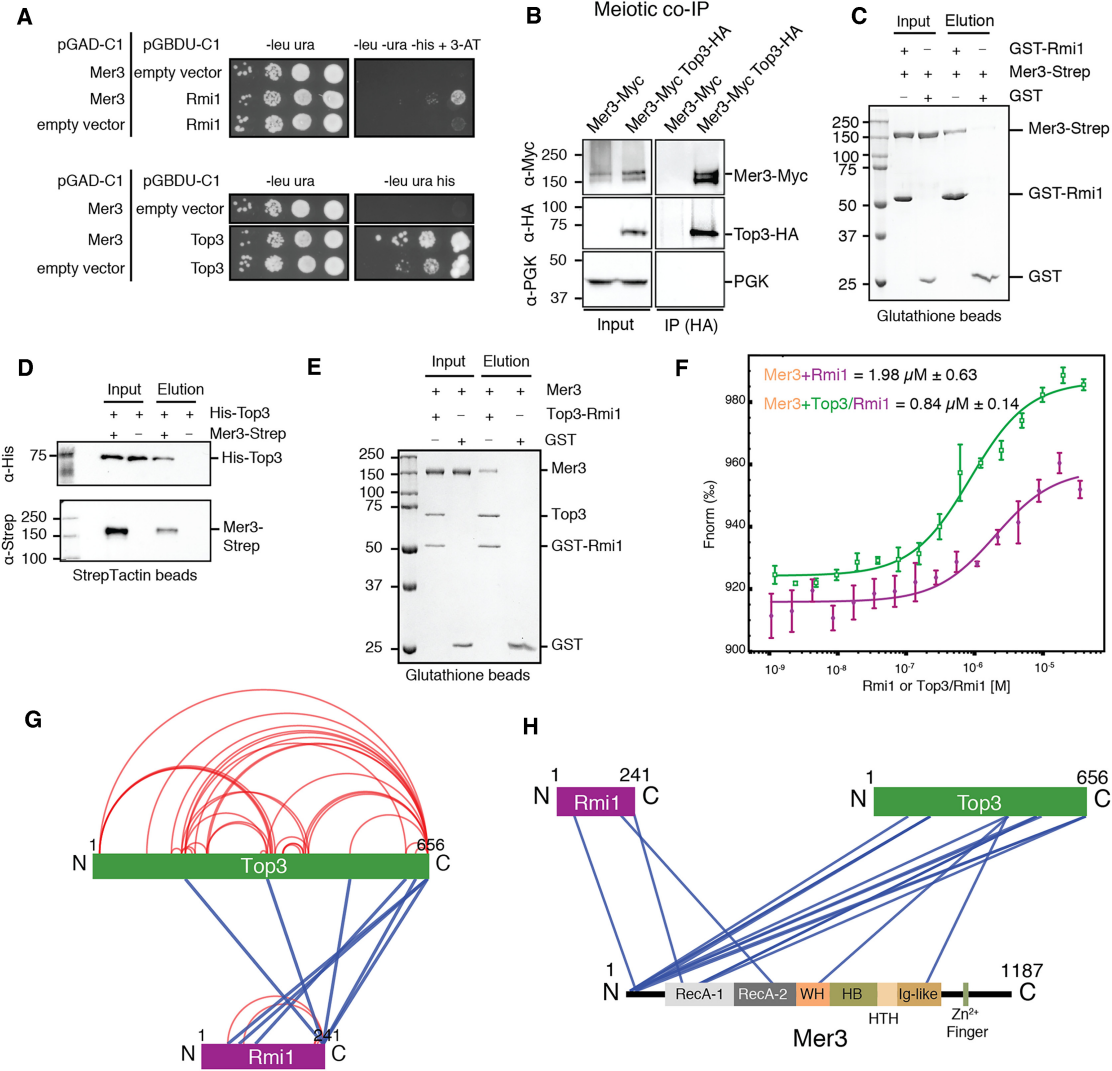
Mer3 binds to Top3 and Rmi1. (**A**) Y2H screen identified a specific interaction between Mer3 and Rmi1 (top panel) and Mer3 and Top3 (bottom panel). (**B**) Meiotic co-IP; after 6 h in sporulation media lysates were precipitated on anti-HA beads. Anti-Myc western blotting was used to detect Myc-tagged Mer3 in the presence (right lane) or absence (left lane) of HA-tagged Top3. (**C**) Glutathione pulldown of recombinant GST-tagged Rmi1 (2.6 μM) against Mer3-Strep (1 μM). GST alone (3.7 μM) was used as a control for non-specific binding to Mer3. (**D**) StrepTactin pulldown of recombinant Mer3-Strep (0.7 μM) against His-Top3 (1.3 μM). Western blotting (anti-Strep and anti-His) was used to confirm the specificity of binding. (**E**) Glutathione pulldown of recombinant GST-tagged Rmi1/Top3 complex (0.8 μM) against Mer3-Strep (0.7 μM). GST alone (3.7 μM) was used as a control for non-specific binding to Mer3. (**F**) Microscale thermophoresis (MST) of Mer3 binding to Rmi1 (purple) or Top3/Rmi1 complex (green). Mer3 was fluorescently labelled and either Rmi1 alone or Top3/Rmi1 titrated against Mer3. Experiments were carried out in triplicate and the *K*_D_ of 1.98 μM (for Rmi1 alone) or 0.84 μM (for Top3/Rmi1) was determined from the fitting curve in the NanoTemper Affinity Analysis v2.3 software (NanoTemper Technologies GmbH). (**G**) Visualisation of Top3/Rmi1 complex XL-MS experiment (using XVis (71)). Intra-chain cross-links are shown in red; inter-chain cross-links in blue. Only high confidence cross-links (score > 50; FDR < 1%) common to two independent datasets are shown. (**H**) Visualisation of Mer3/Top3/Rmi1 complex XL-MS experiment (using XVis (71)). Intra-chain cross-links are not shown for greater clarity. Only high confidence cross-links (score > 50; FDR < 1%) common to two independent datasets are shown.

It was previously inferred that the ZMM proteins must antagonise the anti-crossover activity of Sgs1 helicase (22). However, recent IP-MS studies had not revealed any potential interaction partners of Mer3 that could facilitate this (34). Given that Sgs1 activity is also required for normal CO formation in meiosis (15), we speculated any ZMM interaction that could counteract Sgs1 activity might be transient, and therefore not amenable to proteomics. Thus we made use of a small-scale yeast-two-hybrid screen and evaluated the interaction of Mer3 with several known components of the cross-over pathway. We found that Mer3 can interact with both Rmi1 and Top3 (Figure 3A). Rmi1 and Top3 are both co-factors for Sgs1, promote its helicase activity (61,62) and form a so-called ‘STR complex’ (63). To confirm that Mer3 physically interacts with the Top3/Rmi1 complex in meiosis, we performed a co-immunoprecipitation experiment from *S. cerevisiae* SK1 strain after 6 hours in meiosis. C-terminally tagged Mer3 co-immunoprecipitated with C-terminally HA-tagged Top3 thus confirming that both proteins can associate with one another during meiosis (Figure 3B).

To further study the nature of the Mer3/Top3/Rmi1 interaction we purified both Rmi1 and Top3 as well as a Top3/Rmi1 complex from insect cells (Supplementary Figure S4A). We confirmed the complex formation using these recombinant proteins in an *in vitro* pulldown assay using C-terminally 2xStrepII-tagged Mer3 (Mer3-Strep) and either GST-Rmi1 or His-Top3. We detected an interaction between both Mer3-Strep and GST-Rmi1 (Figure 3C) and His-Top3 (Figure 3D), indicating that Mer3 makes physical contacts with both proteins. To determine whether both interactions are compatible we also carried out a pulldown using Top3/GST-Rmi1 complex. In these combinations, proteins were also interacting indicating that Mer3 interacts with the Top3–Rmi1 complex (Figure 3E).

We characterised the affinity of the Mer3 to Top3/Rmi1 interaction using microscale thermophoresis (MST). Measured *K*_D_ of Mer3 binding to Top3/Rmi1 complex was 844 ± 148 nM (Figure 3F, green). Importantly, when we measured the binding for Rmi1 alone, the binding was weaker (1.99 ± 0.63 μM; Figure 3F, purple) confirming the cooperative nature of the Mer3–Top3–Rmi1 complex assembly.

We used AlphaFold2 to predict the structure of the Top3/Rmi1 complex. Overall confidence in the model quality was very high (Supplementary Figure S4B and C) and the model strongly resembles the experimentally determined structure of the human TopoIIIα-RMI1 complex (RMSD of 1.01Å over 444 residues) (64). To evaluate the AF2 model of Top3/Rmi1, we carried out XL-MS on the purified Top3/Rmi1 complex (Figure 3G). 80% of the high confidence crosslinks were consistent with the model (Supplementary Figure S4D).

XL-MS was also used to study the interaction between the Top3/Rmi1 complex and Mer3 (Figure 3H). Top3 showed the most extensive cross-linking with Mer3, with Top3 cross-links clustering in three regions of Mer3; the N-terminal unstructured region, RecA-1 and the Ig-like domain (Figure 3H). We modelled the Top3/Rmi1 cross-links onto the surface of the Mer3 AF2 model (Supplementary Figure S4E). This revealed that the cross-links cluster on one face of the Mer3 molecule made up of the RecA-1 and Ig-like domains, indicating a likely binding site on Mer3.

### Mer3 interaction with Mlh1/2 is compatible with Top3-Rmi1 binding forming a 5-subunit complex

**Figure 4.**
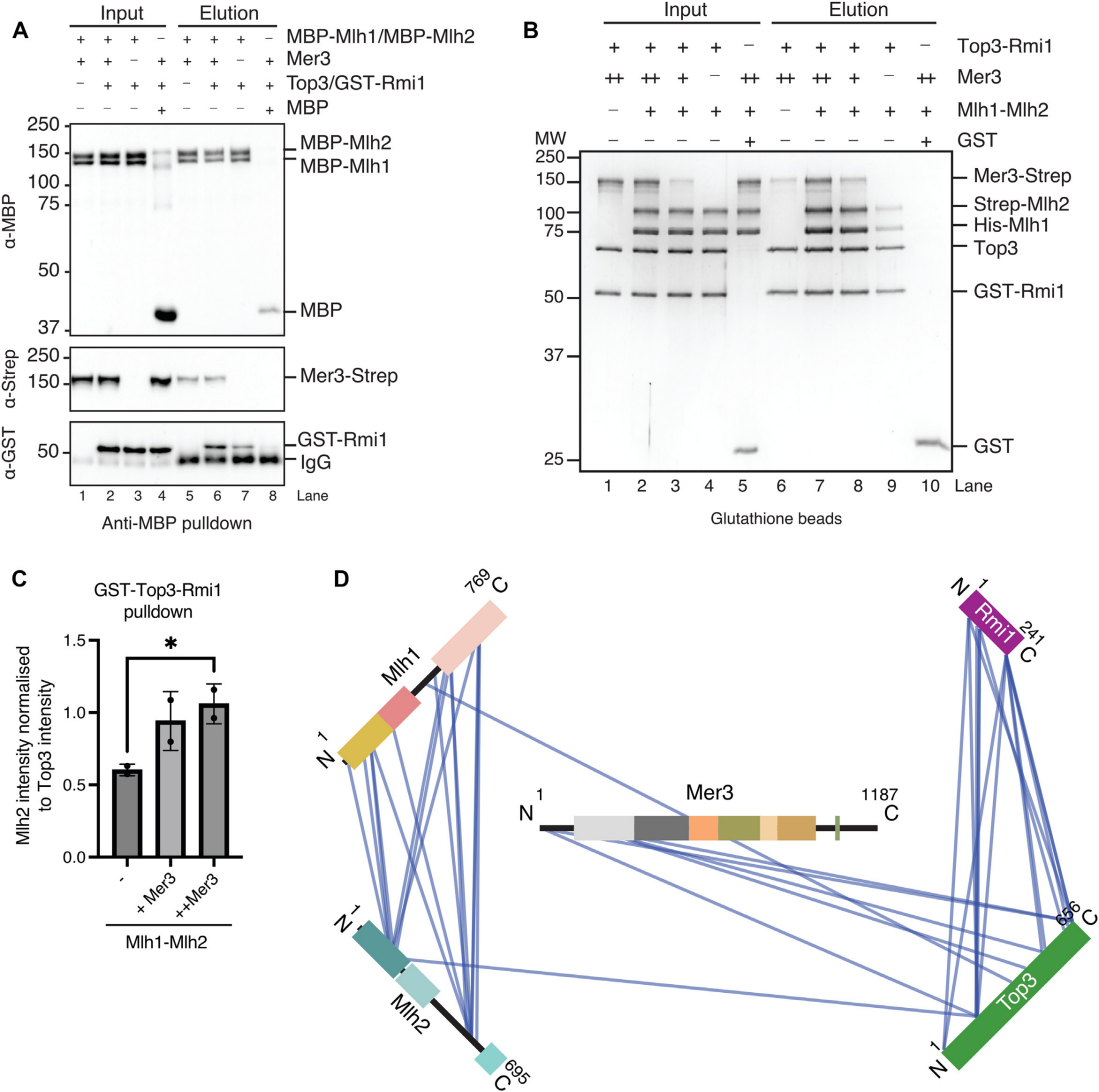
Formation and analysis of a 5-subunit Mer3/Mlh1/Mlh2/Top3/Rmi1 complex. (**A**) Anti-MBP pulldown of recombinant MBP-Mlh1/MBP-Mlh2 complex (0.1 μM) against Mer3-Strep (0.3 μM) (lanes 1 and 5); Mer3-Strep and Top3/GST-Rmi1 (0.3 μM) (lanes 2 and 6); Top3/GST-Rmi1 (lanes 3 and 7). MBP alone (0.9 μM) serves as a control for background binding (lanes 4 and 8). Western blotting was used to identify the differently tagged components as indicated. (**B**) Glutathione pulldown of recombinant Top3/GST-Rmi1 complex (0.8 μM) against Mer3-Strep (0.8 μM) (lanes 1 and 6); Mer3-Strep (0.8 μM), Top3/GST-Rmi1 (0.8 μM) and His-Mlh1/Strep-Mlh2 (0.8 μM) (lanes 2 and 7); Mer3-Strep (0.2 μM), Top3/GST-Rmi1 (0.8 μM) and His-Mlh1/Strep-Mlh2 (lanes 3 and 8), Top3/GST-Rmi1 (0.8 μM) and His-Mlh1/Strep-Mlh2 (0.8 μM) (lanes 4 and 9). GST alone (3.7 μM) serves as a control for background binding (lanes 5 and 10). (**C**) Quantification of the pulldown in B) (two experimental replicates). The intensity of Strep-Mlh2 in each elution lane was calculated as a factor of Top3 intensity. In the presence of Mer3 the intensity of Mlh2 increased (+ Mer3), which was not negatively impacted by a further increased concentration of Mer3 (++ Mer3) (**D**) Visualisation of Mer3/Top3/Rmi1/Mlh1/Mlh2 complex XL-MS experiment (using XVis(71)). Fusion tags and Intra-chain cross-links are not shown for greater clarity. Only high confidence cross-links (score > 50; FDR > 1%) are shown.

Given that the Ig-like domain of Mer3 is involved in binding to Mlh1/Mlh2 complex (31) we tested whether our newly identified binding Top3/Rmi1 by Mer3 is compatible with the Mer3 Mlh1/Mlh2 interaction. We carried out a pulldown using purified Mer3-Strep and Top3/GST-Rmi1 on MBP-Mlh1/MBP-Mlh2 (Figure 4A, lanes 2 and 6). All proteins interacted with each other forming a 5-subunit ‘supercomplex’. Top3/Rmi1 complex interacted with Mlh1/Mlh2 complex also in the absence of Mer3, though at a reduced level (Figure 4A, lanes 3 and 7, compare level of GST-Rmi1 pulled down in lanes 6 and 7), and is indicative of a potential cooperative assembly. We then carried out a pulldown on GST-Rmi1/Top3 using Glutathione beads. Here we used increasing concentrations of Mer3 to determine the effect on Mlh1/Mlh2 recruitment (Figure 4B). We observed that the level of Mlh1/Mlh2 was increased in the presence of Mer3, and did not change as more Mer3 was added to the experiment (Figure 4C), again supporting the idea of a cooperative complex assembly. We again determined the topological structural organisation of the Mer3/Mlh1/Mlh2/Top3/Rmi1 complex using XL-MS, mapping the cross-links that are common between two datasets onto the domain cartoons of the five subunits (Figure 4D). Here, we still observed multiple cross-links between the N-terminal region of Mer3 and Top3, and a single cross-link between Mlh1, Mlh2 and Top3. We compared the two XL-MS datasets independently (Supplementary Figure S5A and B), and found that they showed a broadly similar pattern. We then pooled these two datasets (Supplementary Figure S5C) and mapped the Mlh1/Mlh2 and Top3/Rmi1 cross-links onto the surface of Mer3 (Supplementary Figure S5D). We observe that the majority of the cross-links congregate on a single surface made up of RecA-1, HTH and the Ig-like domains. Again this is indicative of a cooperative assembly involving one face of Mer3, and very likely the unstructured N-terminal 120 amino acids, that interacts with all four components.

### Mer3 and Sgs1 compete with each other for binding to the Top3/Rmi1 complex

**Figure 5.**
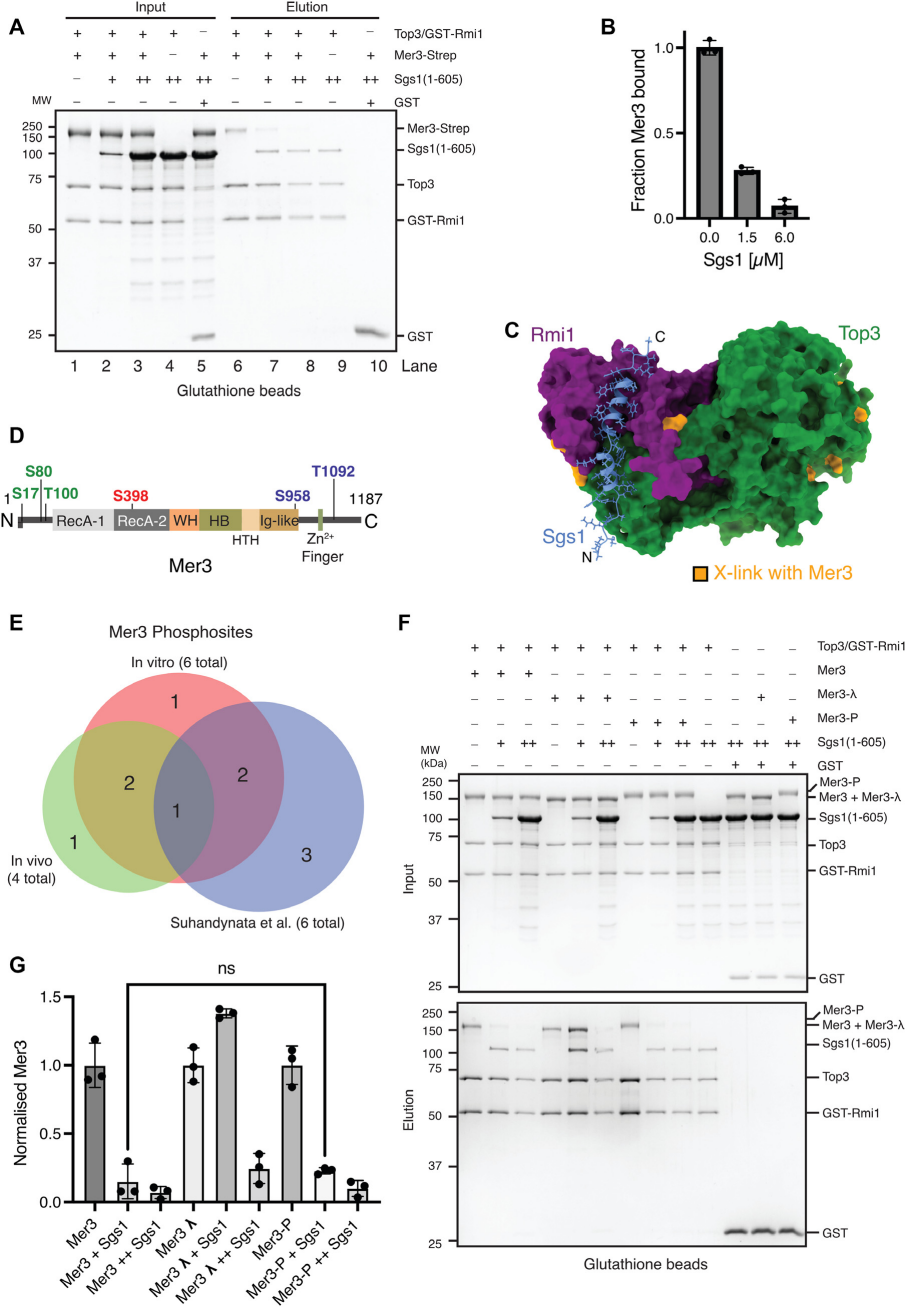
Mer3 and Sgs1 helicases bind competitively for Top3/Rmi1 in a phospho-dependent manner. (**A**) Glutathione pulldown of recombinant Top3/GST-Rmi1 (0.8 μM) against Mer3-Strep (0.8 μM) (lanes 1 and 6), and in increasing concentrations of Sgs1^1–605^ (1.5 μM Sgs1^1–605^ lanes 2 and 7; 6 μM Sgs1^1–605^ lanes 3 and 8). Lanes 4 and 9 show the binding of Sgs1^1–605^ to Top3/GST-Rmi1 in the absence of Mer3-Strep, and lanes 5 and 10 show the background binding of both Mer3-Strep and Sgs1^1-605^ to GST alone (3.7 μM). (**B**) Quantification of lanes 6, 7 and 8 from A). Circles are measurements from three independent experiments, error bars show the SD of these three measurements. (**C**) AF2 structure of the Top3/Rmi1/Sgs1^N^ complex. Surface representation of Top3 (green) and Rmi1 (purple) and a cartoon representation of Sgs1 (blue). The N-terminal helix of Sgs1 (residues 9–34) is predicted to bind to a cleft formed by both Top3 and Rmi1. Residues of Mer3 that cross-link to Top3 and Rmi1 are coloured orange. (**D**) Domain representation of Mer3 showing the six high-confidence phosphosites identified. Green are those sites also found in our *in vivo* data, blue are those sites previously found in Mer3 (66), and red is the one site that is apparently unique to recombinant Mer3 from insect cells. (**E**) Venn diagram showing overlap of phosphosites detected from recombinant Mer3 purified from insect cells (pink), from our *in vivo* phosphosites study (green) and from previous work (blue) (66). (**F**) Glutathione pulldown, carried out in an equivalent manner to A), but using three different versions of Mer3-Strep (each 0.8 μM). Mer3, purified as previously; Mer3-λ, treated with λ-phosphatase; Mer3-P, purified from insect cells treated with okadaic acid. (**G**) Quantification of (F) from three independent replicate pulldowns.

Sgs1 helicase, together with Top3/Rmi1 complex disassemble recombination intermediates and prevent crossover formation. Abolishing the interaction however reduces the activity of both Sgs1 and the Top3/Rmi1 complex(61,65). Given that both Mer3 and Sgs1 are helicases with some structural similarity we asked whether Mer3 competes with Sgs1 for interaction with Top3/Rmi1 complex. We performed a competitive pulldown where we tested whether increasing amounts of Sgs1 can outcompete Mer3 bound to Top3/Rmi1. In this assay, we used only the N-terminal fragment of Sgs1(1–605) that is known to interact with Top3/Rmi1(8) due to our difficulty in obtaining suitable stable full-length recombinant Sgs1 (Figure 5A). Triplicates of the same experiment showed a reproducible competition (Figure 5B). We also carried out the same experiment with a shorter fragment of Sgs1(1–107), containing the previously identified minimum binding region (8) which showed a similar effect (Supplementary Figure S6A).

We were able to create a high-confidence prediction of an N-terminal fragment of Sgs1 bound to Top3/Rmi1 (Figure 5C and Supplementary Figure S6B). When we mapped the residues of Top3–Rmi1 that cross-link to Mer3 we found that these residues form two clusters, one on the N-terminus of Top3, and the second around the predicted Sgs1 binding site (Figure 5C). In light of the potential competitive interaction between Sgs1 and Mer3 for the Top3/Rmi1 complex, we quantitated the affinity of Mer3 binding to Top3/Rmi1 by MST, which we determined to be 478 ± 205 nM (Supplementary Figure S6C)

Given that Sgs1 was able to displace Mer3 from Top3/Rmi1 at relatively low concentrations, and that the measured binding affinity of Sgs1 for Top3/Rmi1 was higher than for Mer3 and Top3/Rmi1 we asked whether the interaction might be modulated through post-translational modifications. We took advantage of the insect cell expression of Mer3 to create three variants. Hyperphosphorylated Mer3 (Mer3-P), was purified from insect cells treated with okadaic acid; ‘normal’ phosphorylated Mer3 was purified from untreated insect cells; dephosphorylated Mer3 (Mer3-λ), was purified from untreated insect cells and subjected to lambda-phosphatase treatment.

Using mass spectrometry we identified 6 high-confidence *in vitro* phosphorylation sites S17, S80, T100, S398, S958 and T1092 (Figure 5D, Supplementary Figure S7A). These high-confidence sites were found in the ‘normal’ Mer3 from insect cells, were increased in the hyperphosphorylated Mer3 (Mer3-P) and were reduced in the dephosphorylated Mer3 (Mer3-λ) (Supplementary Figure S7B). To confirm the physiological relevance of these six sites we enriched for phosphorylated peptides from *S. cerevisiae* SK1 meiotic lysate (6 h in sporulation medium) and subjected triplicate samples to MS analysis. We identified four high-confidence phosphosites, three of which (S17, S80 and T100) were common to the six *in vitro* sites (Supplementary Figure S7C). We further analysed previously described phosphosites of Mer3 (66), which confirmed the presence of two more of the *in vitro* sites in meiosis (summarised in Figure 5E).

We carried out a pulldown experiment, similar to that shown in Figure 5A, except making use of three differentially phosphorylated Mer3 samples (Mer3, Mer3-λ and Mer3-P). Mer3-λ showed far more resistance to Sgs1 competition, whereas both Mer3 and Mer3-P could be more easily displaced from Top3–Rmi1 (Figure 5F and G). Curiously, we observed a small but consistent increase in the amount of Mer3-λ pulled-down on GST-Rmi1/Top3 in the presence of the lower concentration of Sgs1, even when normalised against the signal for Top3 in the pulldown, which might suggest a weak interaction between Sgs1 and Mer3-λ.

### Mer3 inhibits D-loop disassembly mediated by the Sgs1/Top3/Rmi1 complex and Sgs1 alone

**Figure 6.**
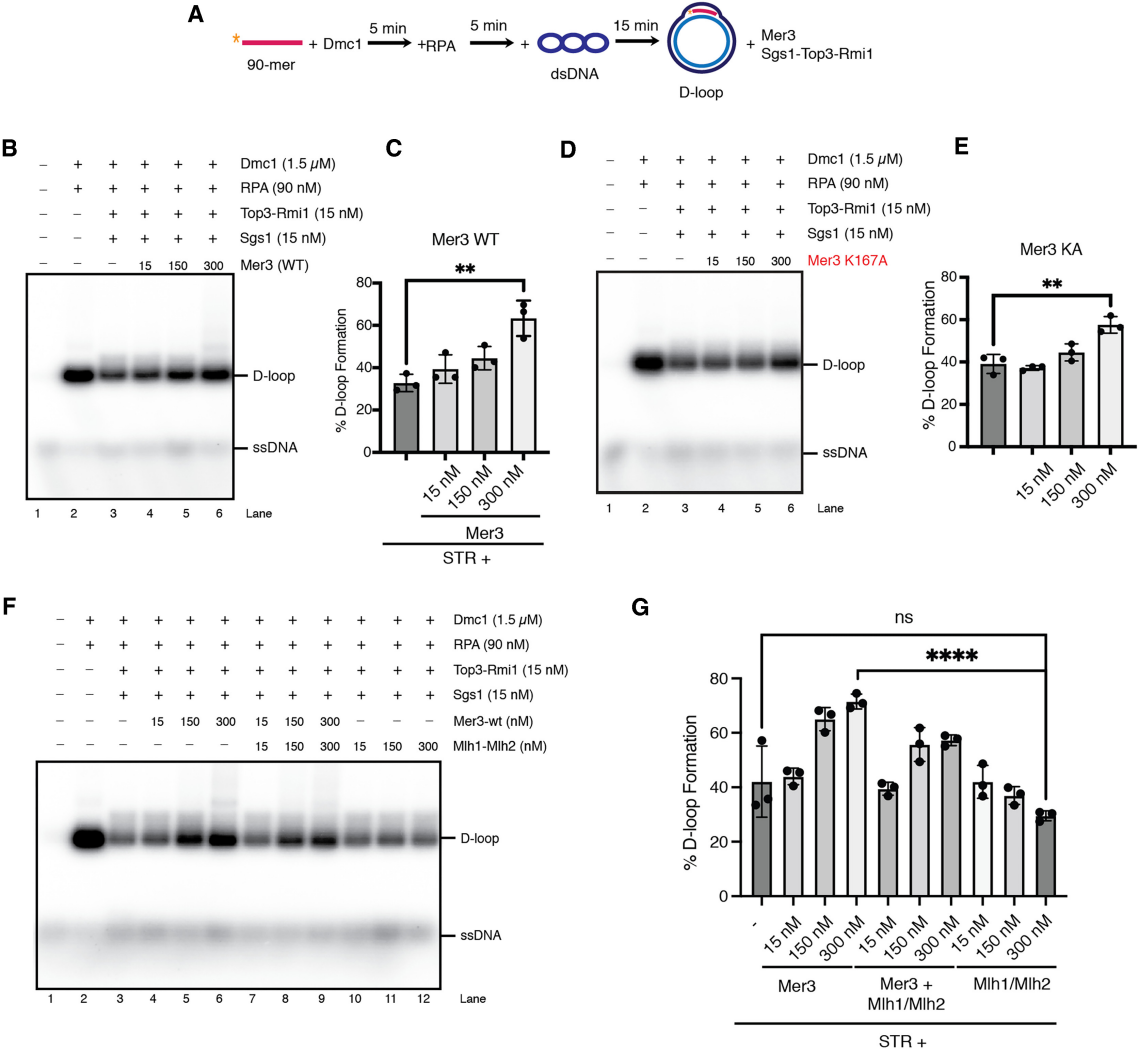
Mer3 protects D-loops from disassembly by STR complex. (**A**) Schematic of the D-loop disassembly experiment. 5′ radio-labelled 90-mer ssDNA was incubated in the presence of Dmc1, followed by RPA, followed by a 15-minute incubation with circular dsDNA to form initial ‘D-loop’ like DNA repair intermediates. These D-loops were then further incubated for 10 minutes in the presence of STR complex, or STR complex with Mer3 or Mlh1/Mlh2. (**B**) Image of samples run on a 0.9% agarose gel. Lane 1 shows radiolabelled ssDNA in the absence of Dmc1 or RPA. Lane 2 shows the formation of D-loops in the presence of Dmc1 and RPA but in the absence of STR or Mer3. Lane 3 shows the effect of the STR complex on D-loops. Lanes 4–6 shows the effect of increasing wild-type Mer3 concentration on the formation of D-loops. (**C**) Quantification of experiments as shown in B). The formation of D-loops in the absence of STR was taken to be 100% formation. Solid circles show values measured from three independent experiments. Error bars show the SD from these three experiments. An unpaired t-test was used to determine statistical significance of the experiments in lanes 3 and 6. (**D**) Image of samples run on a 0.9% agarose gel. Lane 1 shows radiolabelled ssDNA in the absence of Dmc1 or RPA. Lane 2 shows the formation of D-loops in the absence of STR or Mer3^KA^. Lane 3 shows the effect of the STR complex on D-loops. Lanes 4–6 shows the effect of increasing concentration of ATPase dead Mer3 mutant on the formation of D-loops. (**E**) Quantification of experiments as shown in D). The formation of D-loops in the absence of STR was taken to be 100% formation. Solid circles show values measured from three independent experiments. Error bars show the SD from these three experiments. An unpaired t-test was used to determine statistical significance of the experiments in lanes 2 and 5. (**F**) Image of samples run on a 0.9% agarose gel. Lane 1 shows radiolabelled ssDNA in the absence of Dmc1 or RPA. Lane 2 shows the formation of D-loops in the absence of STR, Mer3 or Mlh1/Mlh2. Lane 3 shows the effect of the STR complex on D-loops. Lanes 4–6 show the effect of increasing wild-type Mer3 concentration on the formation of D-loops. Lanes 7–9 show the effect of increasing wild-type Mer3 and Mlh1/Mlh2 complex concentration on the formation of D-loops. Lanes 10–12 show the effect of increasing Mlh1/Mlh2 complex concentration on the formation of D-loops. (**G**) Quantification of experiments as shown in F). The formation of D-loops in the absence of STR was taken to be 100% formation. Solid circles show values measured from three independent experiments. Error bars show the SD from these three experiments. An unpaired *t*-test was used to determine statistical significance of the experiments in lanes 3 and 12 and lanes 6–12.

Given that Mer3 potentially affects the activity of the STR complex, we reconstituted D-loop formation using yeast meiosis-specific recombinase Dmc1 and RPA complex (Figure 6A). Radioactively labelled ssDNA (90-mer) was first incubated with Dmc1 recombinase to form a presynaptic filament. After short incubation with RPA, D-loop formation was initiated by addition of supercoiled plasmid DNA. Then, STR complex (15 nM) was added to the indicated reactions which resulted in robust disruption of the D-loop after 10 min of incubation (30% of relative D-loop yield formed in the absence of STR) (Figure 6B and C). Interestingly, increasing concentrations of Mer3 were able to inhibit the D-loop disassembly by STR complex. 20-fold excess of Mer3 (300 nM) over Sgs1 resulted in a 70% relative yield of D-loop (compared to 30% in the absence of Mer3). Our model makes the assumption that it is the protein binding activity of Mer3, rather than the helicase activity, that inhibits the STR complex. To test this, we made use of an ATP hydrolysis deficient mutant of Mer3, Mer3 K167A. Quantification of triplicate D-loop assays revealed that Mer3^K167A^ showed the same inhibition of STR complex as wild type Mer3 (Figure 6D and E).

Since Mlh1/Mlh2 potentially contributes to a cooperative assembly with Mer3 and Top3/Rmi1 we asked whether the addition of Mlh1/Mlh2 influences the D-loop protection activity of Mer3. Addition of Mlh1/Mlh2 to the Mer3-STR assay did not improve Mer3 mediated D-loop protection, indeed there was a mild reduction in the Mer3 mediated protection (Figure 6F and G). Since Mer3 is a tight binder of D-loops (31) (Supplementary Figure S1A), we asked whether Mer3 might antagonise STR activity simply by binding to D-loops. To test this, we analysed the effect of Mlh1/Mlh2 alone on STR mediated D-loop disassembly. Mlh1/Mlh2 shows binding to D-loops with a similar affinity as Mer3 (Supplementary Figure S3B), though it was previously reported that Mlh1/Mlh2 is a weaker D-loop binder (31). If the Mer3-effect is simply D-loop binding, we would expect to see some level of protection of D-loops when Mlh1/Mlh2 was added, even if a weaker binder of D-loops than Mer3. Interestingly, we saw no effect on STR mediated D-loop disassembly with the addition of Mlh1/Mlh2 complex (Figure 6F and G).

### Mer3 specifically protects Dmc1-mediated D-loops

**Figure 7.**
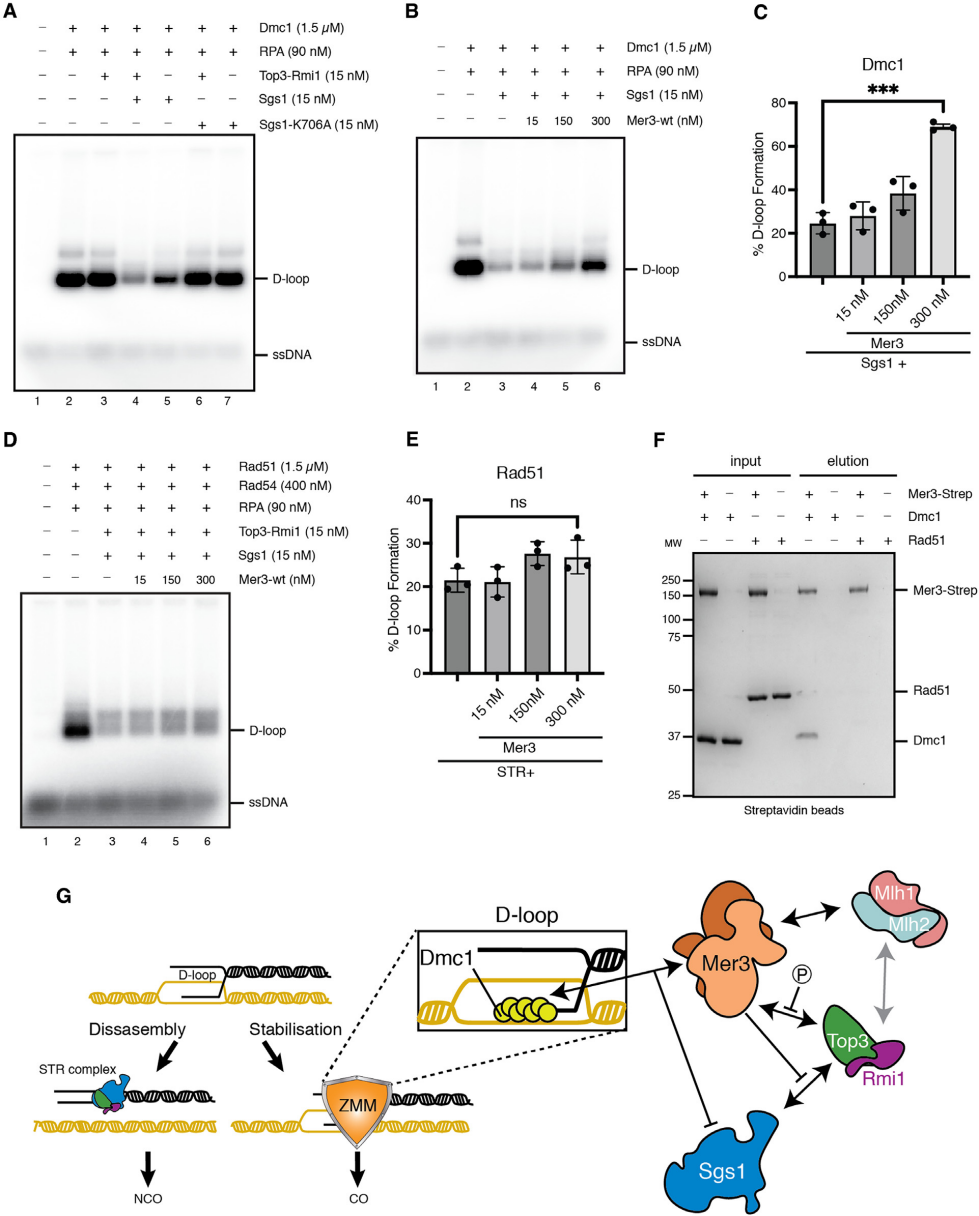
Mer3 protects Dmc1 mediated D-loops from disassembly by Sgs1. (**A**) Image of samples run on a 0.9% agarose gel. Lane 1 shows radiolabelled ssDNA in the absence of Dmc1 or RPA. Lane 2 shows the formation of D-loops in the absence of Top3-Rmi1, Sgs1 or STR. Lane 3 shows the effect of Top3-Rmi1 complex on D-loops. Lane 4 shows the effect of STR complex on D-loops. Lane 5, the effect of Sgs1 alone on D-loops, lanes 6 and 7 are controls with the catalytically inactive Sgs1 helicase (K706A). (**B**) Image of samples run on a 0.9% agarose gel. Lane 1 shows radiolabelled ssDNA in the absence of Dmc1 or RPA. Lane 2 shows the formation of D-loops in the absence of Sgs1 or Mer3. Lane 3 shows the effect of Sgs1 on D-loops. Lanes 4–6 show the effect on D-loops through an increasing concentration of Mer3 (15, 150 and 300 nM). (**C**) Quantification of experiments as shown in B). The formation of D-loops in the absence of Sgs1 was taken to be 100% formation. Solid circles show values measured from three independent experiments. Error bars show the SD from these three experiments. An unpaired t-test was used to determine statistical significance of the experiments in lanes 3 and 6 (*p* = 0.0001). (**D**) Image of samples run on a 0.9% agarose gel. Lane 1 shows radiolabelled ssDNA in the absence of Rad51, Rad54 or RPA. Lane 2 shows the formation of D-loops in the absence of the STR complex or Mer3. Lane 3 shows the effect of the STR complex on D-loops. Lanes 4–6 show the effect on D-loops through an increasing concentration of Mer3 (15, 150 and 300 nM). (**E**) Quantification of experiments as shown in D). The formation of D-loops in the absence of the STR complex was taken to be 100% formation. Solid circles show values measured from three independent experiments. Error bars show the SD from these three experiments. An unpaired t-test was used to determine statistical significance of the experiments in lanes 3 and 6 (*p* = 0.12). (**F**) Streptavidin pulldown of recombinant Mer3-Strep (0.7 μM) against His-Dmc1 (3.2 μM) or Rad51 (2.8 μM). (**G**) Summary of findings. Mer3 binds to Dmc1, and this interaction is required to protect D-loops from disassembly by Sgs1. Mer3 can form dimers, at higher concentrations. Mer3 interacts with Mlh1/Mlh2 and with Top3/Rmi1, and there is a weak interaction between Top3/Rmi1 and Mlh1/Mlh2, thus forming a cooperative 5-subunit complex. Mer3 competes with Sgs1 for binding to Top3/Rmi1, and this competition, and presumably the binding of Mer3 to Top3/Rmi1, is modulated by phosphorylation.

Previous work had shown that Sgs1 required both Top3 and Rmi1 to disassemble Rad51-mediated D-loops *in vitro* (67). First, we asked whether Sgs1 alone could disassemble Dmc1-mediated D-loops. While we observed the strongest D-loop disassembly with the STR complex, we also observed robust D-loop disassembly with Sgs1 alone (Figure 7A). To rule out a potential nuclease contamination, we also performed the assay with helicase-dead mutant Sgs1-K706A which had no effect on D-loop assembly. Next, we analyzed whether the addition of Mer3 to a reaction without Top3/Rmi1 would still show protection of D-loops. We observed that Mer3 provided an equivalent level of D-loop protection against Sgs1 disassembly as against STR disassembly, thus strongly suggesting that the competitive binding between Mer3 and Sgs1 for Top3/Rmi1 is not the molecular basis for Mer3 mediated D-loop protection (Figure 7B).

We then asked whether Mer3 might provide protection to the disassembly of Rad51 mediated D-loops by STR complex. It was previously shown that Rad51 alone mediates D-loop formation poorly, therefore we included Rad54 protein in the reactions which is known to promote Rad51 mediated D-loops(68). Interestingly, the addition of Mer3 to the Rad51 mediated D-loop disassembly reactions provided no observable protection of D-loops (Figure 7D and E).

Given the observed specific difference between Mer3 protection of Dmc1 and Rad51 mediated D-loops, we asked whether Mer3 might directly interact with Dmc1. Using recombinant Dmc1 and Rad51 in a pulldown experiment on Mer3-Strep, we observed direct binding of Mer3 to Dmc1 but not to Rad51 (Figure 7F). Taken together we conclude that Mer3 specifically protects Dmc1 mediated D-loops from Sgs1 disassembly.

## DISCUSSION

Mer3 is a highly conserved member of the ZMM group of proteins that promotes meiotic crossover formation. No experimental structure of Mer3 or its functional orthologs have been described to date. Recent breakthroughs in protein structure prediction, combined with previous biochemical work on both Mer3 and other similar helicases has allowed us to undertake a basic structure–function characterisation of Mer3. It should be noted however the AF2 predictions of Mer3 appear to be at least superficially similar to Mer3 models generated using homology modelling (31). More detailed structure-function analysis was compounded by the observation that Mer3 can form homodimers.

Biophysical analysis of purified Mer3 shows dimer formation at high concentrations, and monomers at low concentrations, and that this dimerisation depends upon the N- and C-terminal unstructured regions. We speculate that in the meiotic nucleoplasm Mer3 is monomeric, but that once D-loops start to form through the course of meiotic prophase, Mer3 forms dimers on D-loops (likely aided by binding to Mlh1/Mlh2, see below). Currently we have no structural information on the organisation of the dimer, beyond the identified ‘self’ cross-links from XL-MS (Figure 1F). One interesting possibility, however, is derived from the spliceosomal Brr2 helicase, to which Mer3 is clearly structurally related. Brr2 contains *two* helicase cassettes in the polypeptide, with the C-terminal cassette lacking catalytic activity, but instead mediating protein–protein interactions. As such we might speculate that one Mer3 subunit is in contact with the D-loop, and extends the D-loop as previously suggested (32), and the second mediating protein-protein interactions. *In vitro* DNA binding assays on Mer3 revealed a similar affinity for D-loops as previously reported (31), but we also observe binding to ssDNA which was not previously seen. This could be due a different purification strategy for Mer3, combined with substrate sequence variation, and.or fluorescent vs. radiolabeled substrates.

One previously identified protein interaction of Mer3 is with the MutLβ complex Mlh1/Mlh2(31). We characterised this interaction further, finding a sub-micromolar *K*_D_, and identifying candidate contact regions and residues through XL-MS. Our data is entirely consistent with the previous work from the Borde laboratory on Mer3 and Mlh1/Mlh2, which showed a role of the Ig-like domain of Mer3 in binding to Mlh1/Mlh2(31). In addition we identify the 1st RecA-like domain of Mer3 plus the N-terminal unstructured region, as an additional potential binding interface with Mlh1/Mlh2. A new protein-protein interaction that we identify is between Mer3 and Top3/Rmi1. We measured the binding affinity to be ∼2-fold less than Mer3 to Mlh1/Mlh2, which might partly explain why this interaction was not previously identified with proteomic approaches in both directions(34,69). We also found that, while the interactions with Top3/Rmi1 and Mlh1/Mlh2 appear to utilise the same surface of Mer3, they are compatible (and likely cooperative) with one another. As such we speculate that Mer3 and Mlh1/Mlh2 cooperate in the context of meiotic D-loops to bind to Top3/Rmi1.

What is the function of Mer3 binding to Top3/Rmi1? We found that Mer3 binds competitively with the Sgs1 helicase (a functional ortholog of BLM helicase) to Top3/Rmi1, disrupting the formation of the STR complex. By combining XL-MS data with an AF2 model of the Sgs1/Top3/Rmi1 structure, we suggest that Mer3 binds to the same cleft on Top3/Rmi1 that is occupied by an N-terminal alpha-helix of Sgs1. Phosphoproteomics of both meiotic lysates and recombinant Mer3 identified several phosphorylation sites. Removal of these sites by lambda-phosphatase reduces the ability of Mer3 to displace Sgs1 from Top3/Rmi1, suggesting that this interaction is subject to phosphoregulation. Further detailed work is required to dissect the function of the novel phosphosites identified in the N-terminal unstructured region of Mer3. While by no means definitive, our data hint at a mechanism by which an increase in cellular kinase activity serves to disrupt the Mer3/Top3/Rmi1 complex, and potentially allow Sgs1 to bind (Figure 5F). This regulated interaction is consistent with the observation that while Sgs1 is an ‘anti-crossover’ helicase, its functionality is also required for normal crossover formation. Therefore we suggest that the Mer3 binding to Top3/Rmi1 is transient, and possibly only at a limited number of loci within the genome.

In a D-loop disassembly assay we find that titrating Mer3 against STR complex reduces the level of D-loop disassembly, thus indicating a Mer3 mediated protection of D-loops.The helicase activity of Mer3 is not required for D-loop ‘protection’ and another D-loop binder, in this case Mlh1/Mlh2 complex, does not protect D-loops from STR complex mediated disassembly. One surprising observation is that the combination of Mlh1/Mlh2 with Mer3 does not provide additional protection to D-loops, indeed the addition of Mlh1/Mlh2 seems to partly mitigate the Mer3 mediated protection.

We considered that the most parsimonious solution was that the presence of Mer3 would disrupt the STR complex and thus protect D-loops through a reduction in Sgs1 activity. However, we also observed Sgs1 could disassemble Dmc1-mediated D-loops in the absence of Top3/Rmi1, and that Mer3 still ‘protected’ D-loops under these conditions. We found that the key factor in Mer3 mediated protection of D-loops was the recombinase. While Mer3 was able to protect Dmc1-formed D-loops, it did not protect Rad51-formed D-loops from Sgs1 mediated disassembly. We found that recombinant Mer3 could bind directly to recombinant Dmc1, but this interaction was not observed for Rad51. Our work suggests that Mer3 would be one of the first ZMM proteins that binds at SEI intermediates, which would be in line with what has been shown previously (31,33).

Several outstanding questions remain to be answered by future experiments. Firstly, when bound to Top3/Rmi1, what interplay is there between the enzymatic activities, i.e. the decatenase and helicase centres? Secondly, besides Mlh1/Mlh2, what other protein factors influence the formation of the Mer3/Top3/Rmi1 complex and potentially contribute to D-loop protection? Thirdly, which kinase is regulating the interaction between Mer3 and Top3/Rmi1, and is there a synergistic effect with phosphorylation of other components in the system (e.g. Sgs1 (17)).

What is the precise molecular basis for the Mer3 mediated protection of D-loops? We propose an initially simple mechanism whereby Mer3 is brought to nascent D-loops that contain Dmc1, through the synergistic effect of Mer3′s affinity for Dmc1 and for D-loops. Here Mer3 is able to protect D-loops against Sgs1 disassembly. Conceivably the interaction of Mer3 with D-loops and Dmc1 is cooperative with Mlh1/Mlh2, which is a known D-loop binder. A detailed mechanistic understanding of the interactions outlined in this study will require separation of function mutants. While our XL-MS data provides some hints as to where these mutants might be made, such a task would be significantly less laborious, and risky, with the benefit of high-resolution structural data, or high-confidence structural predictions.

## DATA AVAILABILITY

The mass spectrometry proteomics data for *in vitro* Mer3 phosphorylation and phospho peptide enrichment of yeast cells have been deposited to the ProteomeXchange Consortium via the PRIDE(70) partner repository with the dataset identifier PXD039839. XL-MS datasets are available as supplementary data.

## Supplementary Material

gkad175_Supplemental_Files

## References

[1] Lam I. , Keeney S. Mechanism and regulation of meiotic recombination initiation. Cold Spring Harb. Perspect. Biol. 2014; 7:a016634. 25324213 10.1101/cshperspect.a016634 PMC4292169

[2] Yadav V.K. , Claeys Bouuaert C. Mechanism and control of meiotic DNA double-strand break formation in *S. cerevisiae*. Front. Cell Dev. Biol. 2021; 9:642737. 33748134 10.3389/fcell.2021.642737 PMC7968521

[3] Humphryes N. , Hochwagen A. A non-sister act: recombination template choice during meiosis. Exp. Cell. Res. 2014; 329:53–60. 25158281 10.1016/j.yexcr.2014.08.024 PMC4561180

[4] LaRocque J.R. , Stark J.M., Oh J., Bojilova E., Yusa K., Horie K., Takeda J., Jasin M. Interhomolog recombination and loss of heterozygosity in wild-type and Bloom syndrome helicase (BLM)-deficient mammalian cells. Proc. Natl. Acad. Sci. U.S.A. 2011; 108:11971–11976. 21730139 10.1073/pnas.1104421108 PMC3141969

[5] Bernstein K.A. , Gangloff S., Rothstein R. The RecQ DNA helicases in DNA repair. Annu. Rev. Genet. 2010; 44:393–417. 21047263 10.1146/annurev-genet-102209-163602 PMC4038414

[6] Johnson F.B. , Lombard D.B., Neff N.F., Mastrangelo M.A., Dewolf W., Ellis N.A., Marciniak R.A., Yin Y., Jaenisch R., Guarente L. Association of the Bloom syndrome protein with topoisomerase iiialpha in somatic and meiotic cells. Cancer Res. 2000; 60:1162–1167. 10728666

[7] Mullen J.R. , Nallaseth F.S., Lan Y.Q., Slagle C.E., Brill S.J. Yeast Rmi1/Nce4 controls genome stability as a subunit of the Sgs1-Top3 complex. Mol. Cell. Biol. 2005; 25:4476–4487. 15899853 10.1128/MCB.25.11.4476-4487.2005 PMC1140617

[8] Bennett R.J. , Noirot-Gros M.-F., Wang J.C. Interaction between Yeast Sgs1 Helicase and DNA topoisomerase III *. J. Biol. Chem. 2000; 275:26898–26905. 10862619 10.1074/jbc.M003137200

[9] van Brabant A.J. , Ye T., Sanz M., German J.L. III, Ellis N.A., Holloman W.K Binding and melting of D-loops by the Bloom syndrome helicase. Biochemistry. 2000; 39:14617–14625. 11087418 10.1021/bi0018640

[10] Bachrati C.Z. , Borts R.H., Hickson I.D. Mobile D-loops are a preferred substrate for the Bloom's syndrome helicase. Nucleic Acids Res. 2006; 34:2269–2279. 16670433 10.1093/nar/gkl258 PMC1456333

[11] Bizard A.H. , Hickson I.D. The dissolution of double Holliday junctions. Cold Spring Harb. Perspect. Biol. 2014; 6:a016477. 24984776 10.1101/cshperspect.a016477 PMC4067992

[12] Amin A.D. , Chaix A.B.H., Mason R.P., Badge R.M., Borts R.H. The roles of the *Saccharomyces cerevisiae* RecQ helicase SGS1 in meiotic genome surveillance. PLoS One. 2010; 5:e15380. 21085703 10.1371/journal.pone.0015380 PMC2976770

[13] De Muyt A. , Jessop L., Kolar E., Sourirajan A., Chen J., Dayani Y., Lichten M. BLM helicase ortholog Sgs1 is a central regulator of meiotic recombination intermediate metabolism. Mol. Cell. 2012; 46:43–53. 22500736 10.1016/j.molcel.2012.02.020 PMC3328772

[14] Tang S. , Wu M.K.Y., Zhang R., Hunter N. Pervasive and essential roles of the Top3-Rmi1 decatenase orchestrate recombination and facilitate chromosome segregation in meiosis. Mol. Cell. 2015; 57:607–621. 25699709 10.1016/j.molcel.2015.01.021 PMC4791043

[15] Oh S.D. , Lao J.P., Hwang P.Y.-H., Taylor A.F., Smith G.R., Hunter N. BLM ortholog, Sgs1, prevents aberrant crossing-over by suppressing formation of multichromatid joint molecules. Cell. 2007; 130:259–272. 17662941 10.1016/j.cell.2007.05.035 PMC2034285

[16] Kaur H. , De Muyt A., Lichten M. Top3-Rmi1 DNA single-strand decatenase is integral to the formation and resolution of meiotic recombination intermediates. Mol. Cell. 2015; 57:583–594. 25699707 10.1016/j.molcel.2015.01.020 PMC4338413

[17] Grigaitis R. , Ranjha L., Wild P., Kasaciunaite K., Ceppi I., Kissling V., Henggeler A., Susperregui A., Peter M., Seidel R. et al. Phosphorylation of the RecQ helicase Sgs1/BLM controls its DNA unwinding activity during meiosis and mitosis. Dev. Cell. 2020; 53:706–723. 32504558 10.1016/j.devcel.2020.05.016

[18] Börner G.V. , Kleckner N., Hunter N. Crossover/noncrossover differentiation, synaptonemal complex formation, and regulatory surveillance at the leptotene/zygotene transition of meiosis. Cell. 2004; 117:29–45. 15066280 10.1016/s0092-8674(04)00292-2

[19] Lynn A. , Soucek R., Börner G.V. ZMM proteins during meiosis: crossover artists at work. Chromosome Res. 2007; 15:591–605. 17674148 10.1007/s10577-007-1150-1

[20] Shinohara M. , Oh S.D., Hunter N., Shinohara A. Crossover assurance and crossover interference are distinctly regulated by the ZMM proteins during yeast meiosis. Nat. Genet. 2008; 40:299–309. 18297071 10.1038/ng.83

[21] Hunter N. , Kleckner N. The single-end invasion: an asymmetric intermediate at the double-strand break to double-holliday junction transition of meiotic recombination. Cell. 2001; 106:59–70. 11461702 10.1016/s0092-8674(01)00430-5

[22] Jessop L. , Rockmill B., Roeder G.S., Lichten M. Meiotic chromosome synapsis-promoting proteins antagonize the anti-crossover activity of sgs1. PLoS Genet. 2006; 2:e155. 17002499 10.1371/journal.pgen.0020155 PMC1570379

[23] Storlazzi A. , Gargano S., Ruprich-Robert G., Falque M., David M., Kleckner N., Zickler D. Recombination proteins mediate meiotic spatial chromosome organization and pairing. Cell. 2010; 141:94–106. 20371348 10.1016/j.cell.2010.02.041 PMC2851631

[24] Mercier R. , Jolivet S., Vezon D., Huppe E., Chelysheva L., Giovanni M., Nogué F., Doutriaux M.-P., Horlow C., Grelon M. et al. Two meiotic crossover classes cohabit in Arabidopsis: one is dependent on MER3,whereas the other one is not. Curr. Biol. 2005; 15:692–701. 15854901 10.1016/j.cub.2005.02.056

[25] Wang K. , Tang D., Wang M., Lu J., Yu H., Liu J., Qian B., Gong Z., Wang X., Chen J. et al. MER3 is required for normal meiotic crossover formation, but not for presynaptic alignment in rice. J. Cell Sci. 2009; 122:2055–2063. 19470578 10.1242/jcs.049080

[26] Chen C. , Zhang W., Timofejeva L., Gerardin Y., Ma H. The Arabidopsis ROCK-N-ROLLERS gene encodes a homolog of the yeast ATP-dependent DNA helicase MER3 and is required for normal meiotic crossover formation. Plant J. 2005; 43:321–334. 16045469 10.1111/j.1365-313X.2005.02461.x

[27] Guiraldelli M.F. , Eyster C., Wilkerson J.L., Dresser M.E., Pezza R.J. Mouse HFM1/Mer3 is required for crossover formation and complete synapsis of homologous chromosomes during meiosis. PLos Genet. 2013; 9:e1003383. 23555294 10.1371/journal.pgen.1003383 PMC3605105

[28] Primary Ovarian Insufficiency Collaboration Wang J. , Zhang W., Jiang H., Wu B.-L. Mutations in HFM1 in recessive primary ovarian insufficiency. N. Engl. J. Med. 2014; 370:972–974. 24597873 10.1056/NEJMc1310150

[29] Nakagawa T. , Flores-Rozas H., Kolodner R.D. The MER3 helicase involved in meiotic crossing over is stimulated by single-stranded DNA-binding proteins and unwinds DNA in the 3′ to 5′ direction. J. Biol. Chem. 2001; 276:31487–31493. 11376001 10.1074/jbc.M104003200 PMC3635102

[30] Nakagawa T. , Kolodner R.D. *Saccharomyces cerevisiae* Mer3 is a DNA helicase involved in meiotic crossing over. Mol. Cell. Biol. 2002; 22:3281–3291. 11971962 10.1128/MCB.22.10.3281-3291.2002 PMC133777

[31] Duroc Y. , Kumar R., Ranjha L., Adam C., Guérois R., Md Muntaz K., Marsolier-Kergoat M.-C., Dingli F., Laureau R., Loew D. et al. Concerted action of the MutLβ heterodimer and Mer3 helicase regulates the global extent of meiotic gene conversion. Elife. 2017; 6:e21900. 28051769 10.7554/eLife.21900 PMC5215242

[32] Mazina O.M. , Mazin A.V., Nakagawa T., Kolodner R.D., Kowalczykowski S.C. *Saccharomyces cerevisiae* Mer3 helicase stimulates 3′–5′ heteroduplex extension by Rad51. Cell. 2004; 117:47–56. 15066281 10.1016/s0092-8674(04)00294-6

[33] Pyatnitskaya A. , Borde V., Muyt A.D. Crossing and zipping: molecular duties of the ZMM proteins in meiosis. Chromosoma. 2019; 128:181–198. 31236671 10.1007/s00412-019-00714-8

[34] Vernekar D.V. , Reginato G., Adam C., Ranjha L., Dingli F., Marsolier M.-C., Loew D., Guérois R., Llorente B., Cejka P. et al. The Pif1 helicase is actively inhibited during meiotic recombination which restrains gene conversion tract length. Nucleic Acids Res. 2021; 49:4522–4533. 33823531 10.1093/nar/gkab232 PMC8096244

[35] Altmannova V. , Blaha A., Astrinidis S., Reichle H., Weir J.R. InteBac: an integrated bacterial and baculovirus expression vector suite. Protein Sci. 2021; 30:108–114. 32955754 10.1002/pro.3957 PMC7737779

[36] Weissmann F. , Petzold G., VanderLinden R., Veld P.J.H.I., Brown N.G., Lampert F., Westermann S., Stark H., Schulman B.A., Peters J.-M. biGBac enables rapid gene assembly for the expression of large multisubunit protein complexes. Proc. Natl. Acad. Sci. U.S.A. 2016; 113:E2564 9. 27114506 10.1073/pnas.1604935113 PMC4868461

[37] Bieniossek C. , Imasaki T., Takagi Y., Berger I. MultiBac: expanding the research toolbox for multiprotein complexes. Trends Biochem. Sci. 2012; 37:49–57. 22154230 10.1016/j.tibs.2011.10.005 PMC7127121

[38] Busygina V. , Gaines W.A., Xu Y., Kwon Y., Williams G.J., Lin S.-W., Chang H.-Y., Chi P., Wang H.-W., Sung P. Functional attributes of the Saccharomyces cerevisiae meiotic recombinase Dmc1. DNA Repair (Amst.). 2013; 12:707–712. 23769192 10.1016/j.dnarep.2013.05.004 PMC3732525

[39] Van Komen S. , Macris M., Sehorn M.G., Sung P. Purification and assays of saccharomyces cerevisiae homologous recombination proteins. Methods in Enzymology. 2006; 408:Academic Press 445–463. 16793386 10.1016/S0076-6879(06)08028-1

[40] De Muyt A. , Pyatnitskaya A., Andréani J., Ranjha L., Ramus C., Laureau R., Fernandez-Vega A., Holoch D., Girard E., Govin J. et al. A meiotic XPF-ERCC1-like complex recognizes joint molecule recombination intermediates to promote crossover formation. Genes Dev. 2018; 32:283–296. 29440262 10.1101/gad.308510.117 PMC5859969

[41] Ranjha L. , Anand R., Cejka P. The *Saccharomyces cerevisiae* Mlh1-Mlh3 heterodimer is an endonuclease that preferentially binds to Holliday junctions. J. Biol. Chem. 2014; 289:5674–5686. 24443562 10.1074/jbc.M113.533810 PMC3937642

[42] Pan D. , Brockmeyer A., Mueller F., Musacchio A., Bange T. Simplified protocol for cross-linking mass spectrometry using the MS-cleavable cross-linker DSBU with efficient cross-link identification. Anal. Chem. 2018; 90:10990–10999. 30074391 10.1021/acs.analchem.8b02593

[43] Götze M. , Pettelkau J., Fritzsche R., Ihling C.H., Schäfer M., Sinz A. Automated assignment of MS/MS cleavable cross-links in protein 3D-structure analysis. J. Am. Soc. Mass. Spectrom. 2015; 26:83–97. 25261217 10.1007/s13361-014-1001-1

[44] Schiffrin B. , Radford S.E., Brockwell D.J., Calabrese A.N. PyXlinkViewer: a flexible tool for visualization of protein chemical crosslinking data within the PyMOL molecular graphics system. Protein Sci. 2020; 29:1851–1857. 32557917 10.1002/pro.3902 PMC7380677

[45] Lagerwaard I.M. , Albanese P., Jankevics A., Scheltema R.A. Xlink mapping and AnalySis (XMAS) - smooth integrative modeling in ChimeraX. 2022; bioRxiv doi:29 June 2022, preprint: not peer reviewed 10.1101/2022.04.21.489026.

[46] Evans R. , O’Neill M., Pritzel A., Antropova N., Senior A., Green T., Žídek A., Bates R., Blackwell S., Yim J. et al. Protein complex prediction with AlphaFold-multimer. 2021; bioRxiv doi:10 March 2022, preprint: not peer reviewed 10.1101/2021.10.04.463034.

[47] Janke C. , Magiera M.M., Rathfelder N., Taxis C., Reber S., Maekawa H., Moreno-Borchart A., Doenges G., Schwob E., Schiebel E. et al. A versatile toolbox for PCR-based tagging of yeast genes: new fluorescent proteins, more markers and promoter substitution cassettes. Yeast. 2004; 21:947–962. 15334558 10.1002/yea.1142

[48] Knop M. , Siegers K., Pereira G., Zachariae W., Winsor B., Nasmyth K., Schiebel E. Epitope tagging of yeast genes using a PCR-based strategy: more tags and improved practical routines. Yeast. 1999; 15:963–972. 10407276 10.1002/(SICI)1097-0061(199907)15:10B<963::AID-YEA399>3.0.CO;2-W

[49] Humphrey S.J. , Karayel O., James D.E., Mann M. High-throughput and high-sensitivity phosphoproteomics with the EasyPhos platform. Nat. Protoc. 2018; 13:1897–1916. 30190555 10.1038/s41596-018-0014-9

[50] Cox J. , Mann M. MaxQuant enables high peptide identification rates, individualized p.P.B.-range mass accuracies and proteome-wide protein quantification. Nat. Biotechnol. 2008; 26:1367–1372. 19029910 10.1038/nbt.1511

[51] Borek W.E. , Vincenten N., Duro E., Makrantoni V., Spanos C., Sarangapani K.K., de Lima Alves F., Kelly D.A., Asbury C.L., Rappsilber J. et al. The proteomic landscape of centromeric chromatin reveals an essential role for the Ctf19CCAN complex in meiotic kinetochore assembly. Curr. Biol. 2021; 31:283–296. 33157029 10.1016/j.cub.2020.10.025 PMC7846277

[52] Tyanova S. , Temu T., Sinitcyn P., Carlson A., Hein M.Y., Geiger T., Mann M., Cox J. The Perseus computational platform for comprehensive analysis of (prote)omics data. Nat. Methods. 2016; 13:731–740. 27348712 10.1038/nmeth.3901

[53] Jumper J. , Evans R., Pritzel A., Green T., Figurnov M., Ronneberger O., Tunyasuvunakool K., Bates R., Žídek A., Potapenko A. et al. Highly accurate protein structure prediction with AlphaFold. Nature. 2021; 596:583–589. 34265844 10.1038/s41586-021-03819-2 PMC8371605

[54] Holm L. Using Dali for protein structure comparison. Methods Mol. Biol. 2020; 2112:29–42. 32006276 10.1007/978-1-0716-0270-6_3

[55] Pena V. , Jovin S.M., Fabrizio P., Orlowski J., Bujnicki J.M., Lührmann R., Wahl M.C. Common design principles in the spliceosomal RNA helicase Brr2 and in the Hel308 DNA helicase. Mol. Cell. 2009; 35:454–466. 19716790 10.1016/j.molcel.2009.08.006

[56] Ponting C.P. Proteins of the endoplasmic-reticulum-associated degradation pathway: domain detection and function prediction. Biochem. J. 2000; 351 Pt 2:527–535. 11023840 PMC1221390

[57] Müller M.Q. , Dreiocker F., Ihling C.H., Schäfer M., Sinz A. Cleavable cross-linker for protein structure analysis: reliable identification of cross-linking products by tandem MS. Anal. Chem. 2010; 82:6958–6968. 20704385 10.1021/ac101241t

[58] Ho B. , Baryshnikova A., Brown G.W. Unification of protein abundance datasets yields a quantitative saccharomyces cerevisiae proteome. Cell Syst. 2018; 6:192–205. 29361465 10.1016/j.cels.2017.12.004

[59] Gueneau E. , Dherin C., Legrand P., Tellier-Lebegue C., Gilquin B., Bonnesoeur P., Londino F., Quemener C., Le Du M.-H., Márquez J.A. et al. Structure of the MutLα C-terminal domain reveals how Mlh1 contributes to Pms1 endonuclease site. Nat. Struct. Mol. Biol. 2013; 20:461–468. 23435383 10.1038/nsmb.2511

[60] Ban C. , Junop M., Yang W. Transformation of MutL by ATP binding and hydrolysis: a switch in DNA mismatch repair. Cell. 1999; 97:85–97. 10199405 10.1016/s0092-8674(00)80717-5

[61] Cejka P. , Plank J.L., Bachrati C.Z., Hickson I.D., Kowalczykowski S.C. Rmi1 stimulates decatenation of double Holliday junctions during dissolution by Sgs1-Top3. Nat. Struct. Mol. Biol. 2010; 17:1377–1382. 20935631 10.1038/nsmb.1919 PMC2988882

[62] Kasaciunaite K. , Fettes F., Levikova M., Daldrop P., Anand R., Cejka P., Seidel R. Competing interaction partners modulate the activity of Sgs1 helicase during DNA end resection. EMBO J. 2019; 38:e101516. 31268598 10.15252/embj.2019101516 PMC6601037

[63] Chang M. , Bellaoui M., Zhang C., Desai R., Morozov P., Delgado-Cruzata L., Rothstein R., Freyer G.A., Boone C., Brown G.W. RMI1/NCE4, a suppressor of genome instability, encodes a member of the RecQ helicase/Topo III complex. EMBO J. 2005; 24:2024–2033. 15889139 10.1038/sj.emboj.7600684 PMC1142613

[64] Bocquet N. , Bizard A.H., Abdulrahman W., Larsen N.B., Faty M., Cavadini S., Bunker R.D., Kowalczykowski S.C., Cejka P., Hickson I.D. et al. Structural and mechanistic insight into Holliday-junction dissolution by topoisomerase iiiα and RMI1. Nat. Struct. Mol. Biol. 2014; 21:261–268. 24509834 10.1038/nsmb.2775 PMC4292918

[65] Cejka P. , Plank J.L., Dombrowski C.C., Kowalczykowski S.C. Decatenation of DNA by the *S. cerevisiae* Sgs1-Top3-Rmi1 and RPA complex: a mechanism for disentangling chromosomes. Mol. Cell. 2012; 47:886–896. 22885009 10.1016/j.molcel.2012.06.032 PMC3462259

[66] Suhandynata R.T. , Wan L., Zhou H., Hollingsworth N.M. Identification of putative Mek1 substrates during meiosis in saccharomyces cerevisiae using quantitative phosphoproteomics. PLoS One. 2016; 11:e0155931. 27214570 10.1371/journal.pone.0155931 PMC4877051

[67] Fasching C.L. , Cejka P., Kowalczykowski S.C., Heyer W.-D. Top3-Rmi1 dissolve Rad51-mediated D loops by a topoisomerase-based mechanism. Mol. Cell. 2015; 57:595–606. 25699708 10.1016/j.molcel.2015.01.022 PMC4338411

[68] Petukhova G. , Stratton S., Sung P. Catalysis of homologous DNA pairing by yeast Rad51 and Rad54 proteins. Nature. 1998; 393:91–94. 9590697 10.1038/30037

[69] Wild P. , Susperregui A., Piazza I., Dörig C., Oke A., Arter M., Yamaguchi M., Hilditch A.T., Vuina K., Chan K.C. et al. Network rewiring of homologous recombination enzymes during mitotic proliferation and meiosis. Mol. Cell. 2019; 75:859–874. 31351878 10.1016/j.molcel.2019.06.022 PMC6715774

[70] Perez-Riverol Y. , Bai J., Bandla C., García-Seisdedos D., Hewapathirana S., Kamatchinathan S., Kundu D.J., Prakash A., Frericks-Zipper A., Eisenacher M. et al. The PRIDE database resources in 2022: a hub for mass spectrometry-based proteomics evidences. Nucleic Acids Res. 2021; 50:D543–D552. 10.1093/nar/gkab1038 PMC8728295 34723319

[71] Grimm M. , Zimniak T., Kahraman A., Herzog F. xVis: a web server for the schematic visualization and interpretation of crosslink-derived spatial restraints. Nucleic Acids Res. 2015; 43:W362–W369. 25956653 10.1093/nar/gkv463 PMC4489277

