# Supplementary material for: Biochemical characterisation of Mer3 helicase interactions and the protection of meiotic recombination intermediates
Source: Nucleic Acids Res. 2023 Mar 21;51(9):4363–84. doi: 10.1093/nar/gkad175 (PMC10201424; doi:10.1093/nar/gkad175)
Supplement: gkad175_Supplemental_Files [file gkad175_supplemental_files.zip › Supplementary Data.pdf]

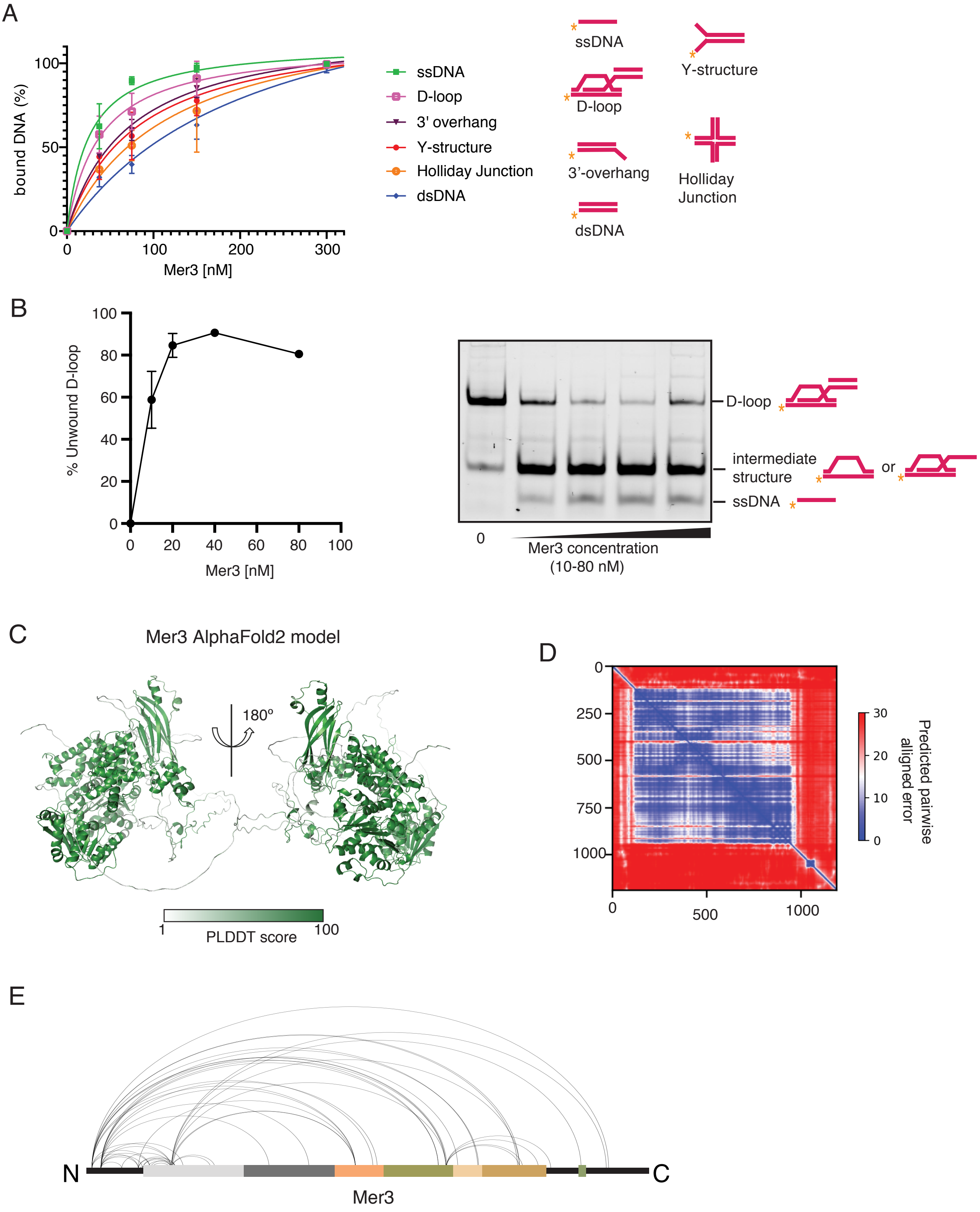

Supplementary Figure 1

Supplementary Figure 1

A) Summary of EMSAs of recombinant Mer3-Strep binding to different DNA substrates (cartoon representations of substrates, right). Mer3 bound to ssDNA and D-loops with the highest affinity.

B) Strand separation activity of Mer3 on D-loop substrate. Graph shows quantification of three independent experiments (example shown right) for strand separation activity.

C) AlphaFold2 model of *S. cerevisiae* Mer3 coloured according to pLDDT score.

D) PAE plot of the AlphaFold2 model of Mer3.

E) Visualisation of Mer3 XL-MS data showing the intrachain cross-links on the domain cartoon of Mer3 (domain cartoon coloured as in Figure 1D.

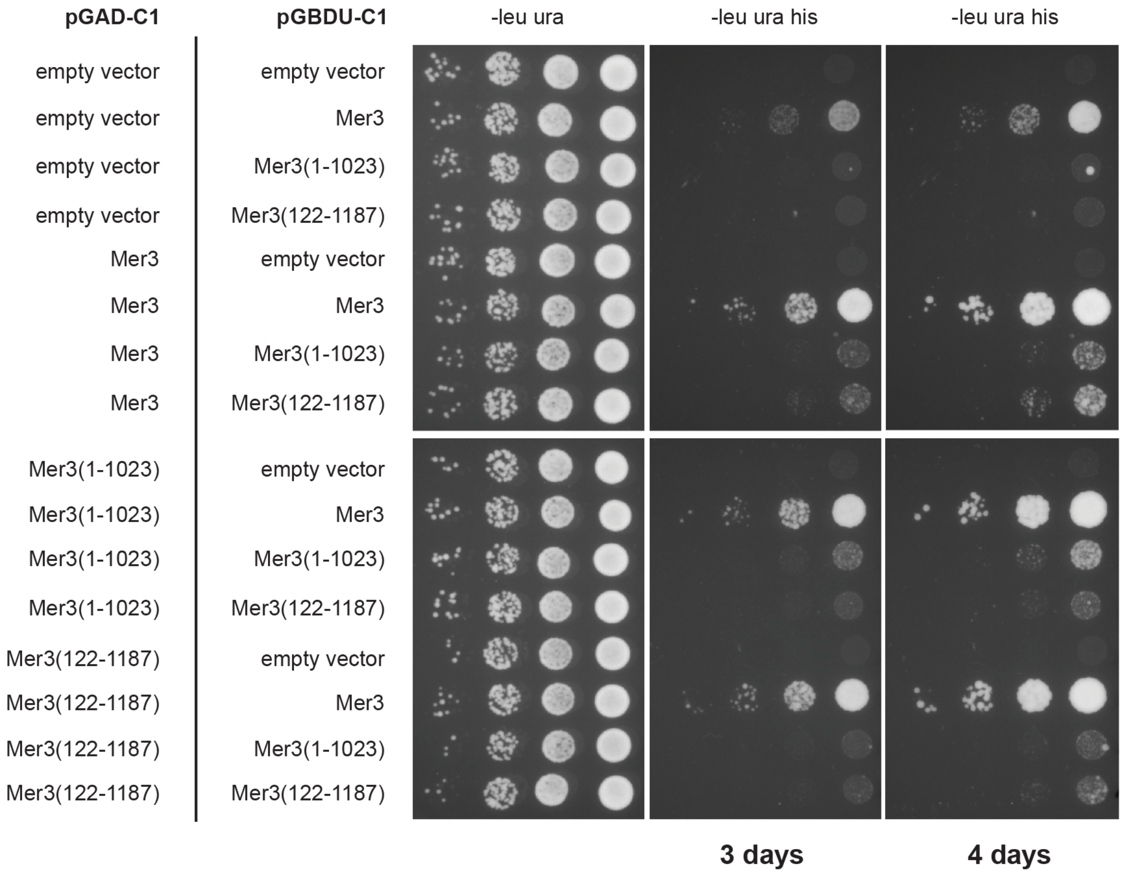

Supplementary Figure 2

Supplementary Figure 2 - Yeast-two-hybrid experiments

The role of the unstructured N- and C-terminal regions in Mer3 self-association was tested in a series of yeast-two-hybrid experiments. Vectors used as indicated in the figure. Images are from a 3 or 4 day incubation at 30ºC on the plates indicated.

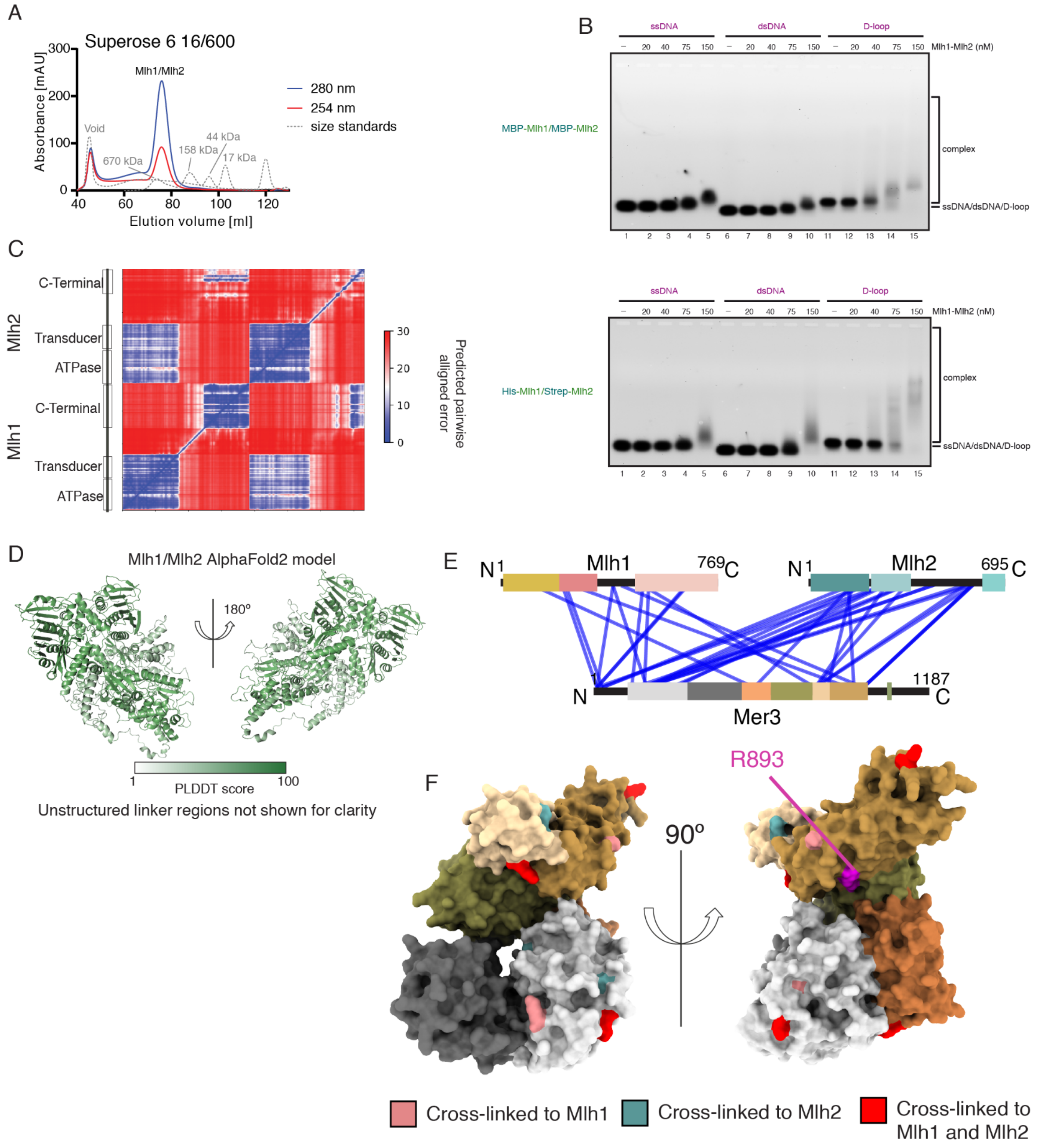

Supplementary Figure 3

Supplementary Figure 3

A) SEC profile of the purification of *S. cerevisiae* Mlh1/Mlh2 showing absorbance at 280 nm (blue) or 260 nm (red). SEC standards include the bio-rad gel filtration standard, and dextran blue as a void marker.

B) DNA binding activity of purified Mlh1-Mlh2 complexes with different affinity tags on various fluorescently labelled DNA substrates.

C) AF2 multimer structure of Mlh1/Mlh2 heterodimer coloured by the pLDDT score.

D) PAE plot of the AF2 multimer prediction of the Mlh1/Mlh2 heterodimer.

E) Merged cross-links from two independent datasets, showing only the interactions between Mer3 and Mlh1 or Mer3 and Mlh2.

F) Surface representation of the AF2 model of Mer3. Domains coloured as in Figure 1D. Residues coloured additionally according to which cross-links detected within the context of the DSBU treated Mer3/Mlh1/Mlh2 complex (Mlh1, pink; Mlh2, blue; both, red). Location of R893 is highlighted as this was previously shown to disrupt the interaction between Mer3 and Mlh1/Mlh2.

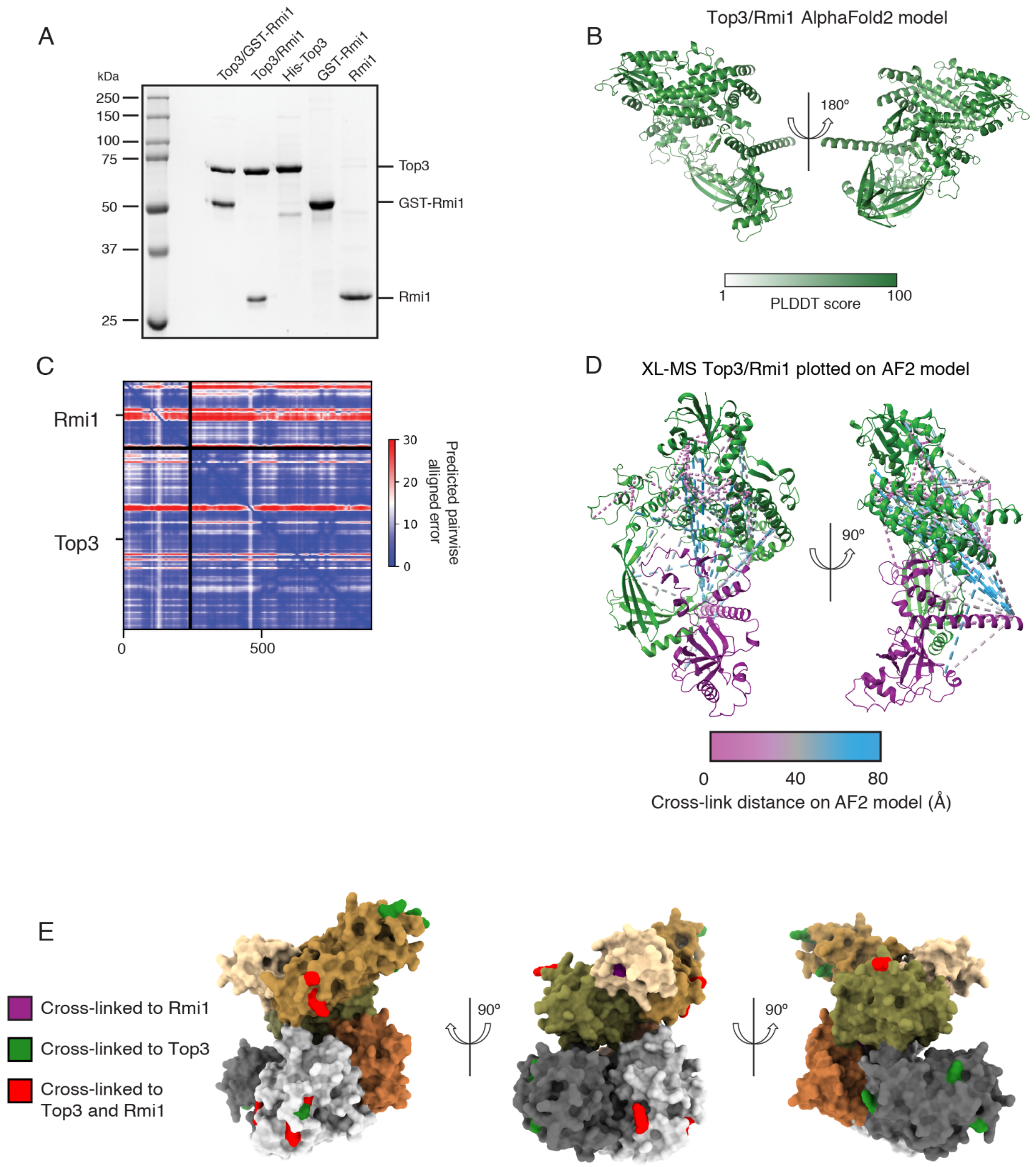

Supplementary Figure 4

Supplementary Figure 4

A) Representative coomassie stained SDS-PAGE from purifications of GST-Rmi1, His-Top3, Top3/GST-Rmi1 and Rmi1.

B) AF2 multimer structure of *S. cerevisiae* Top3/Rmi1 heterodimer coloured by the pLDDT score.

C) PAE plot of the AF2 multimer structure of *S. cerevisiae* Top3/Rmi1 heterodimer.

D) XL-MS data of the Top3/Rmi1 complex plotted onto the AlphaFold2 multimer model using XMAS (62). Cross-links are coloured according to distance.

E) Surface representation of the AF2 model of Mer3. Domains coloured as in Figure 1D. Residues coloured additionally according to which cross-links detected within the context of the DSBU treated Mer3/Top3/Rmi1 complex (Top3, green; Rmi1, purple; both, red).

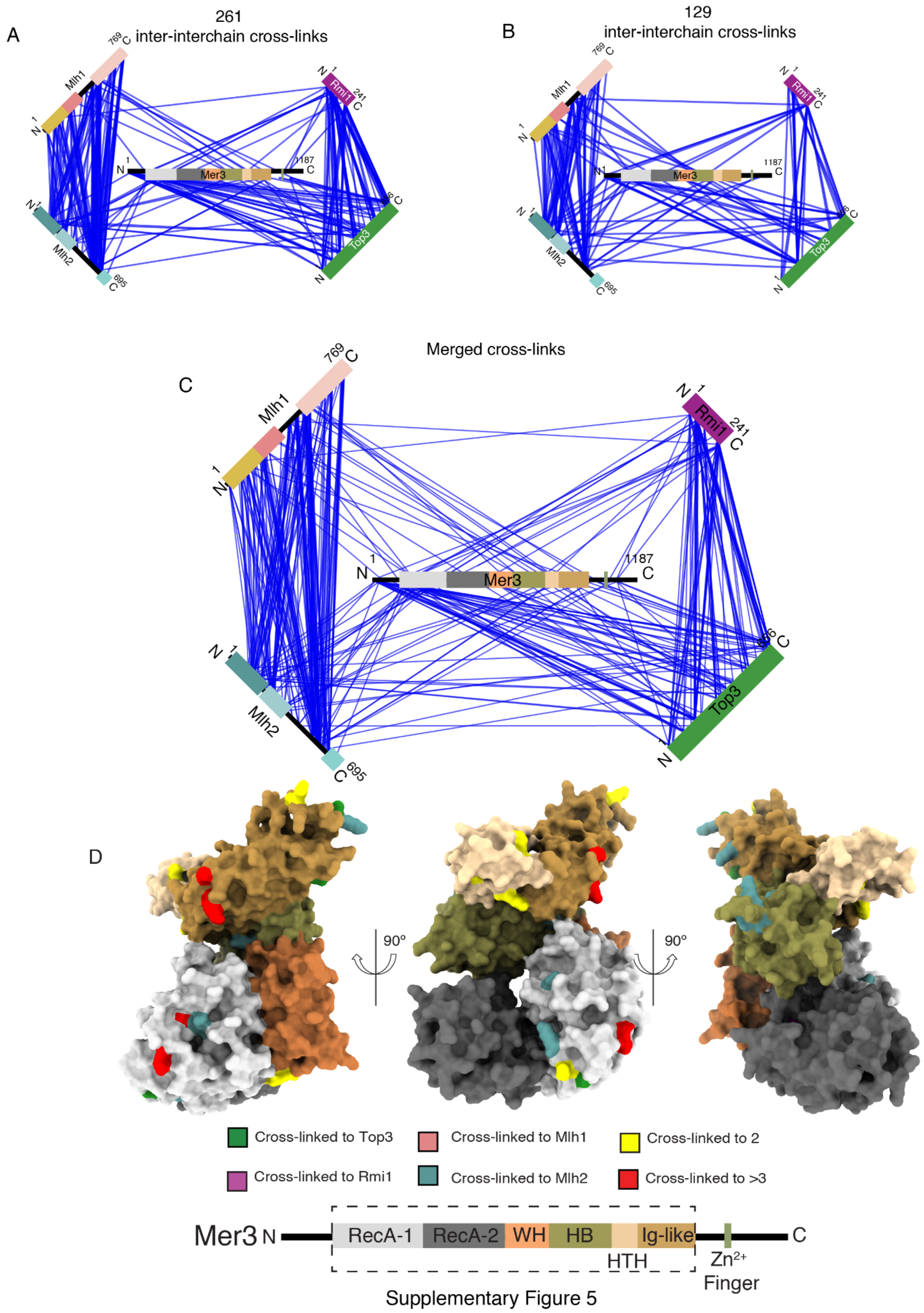

Supplementary Figure 5

Supplementary Figure 5 - XL-MS on 5-subunit complex

A) Interchain cross-links from Dataset #1. Confidence cut offs were applied to achieve a FDR <1%.

B) Interchain cross-links from Dataset #2. Confidence cut offs were applied to achieve a FDR <1%.
C) Merger of cross-links from Dataset #1 and #2 (i.e.shown in A and B).
D) Mapping of merged cross-links onto the surface of the Mer3 Alpha Fold2 model.

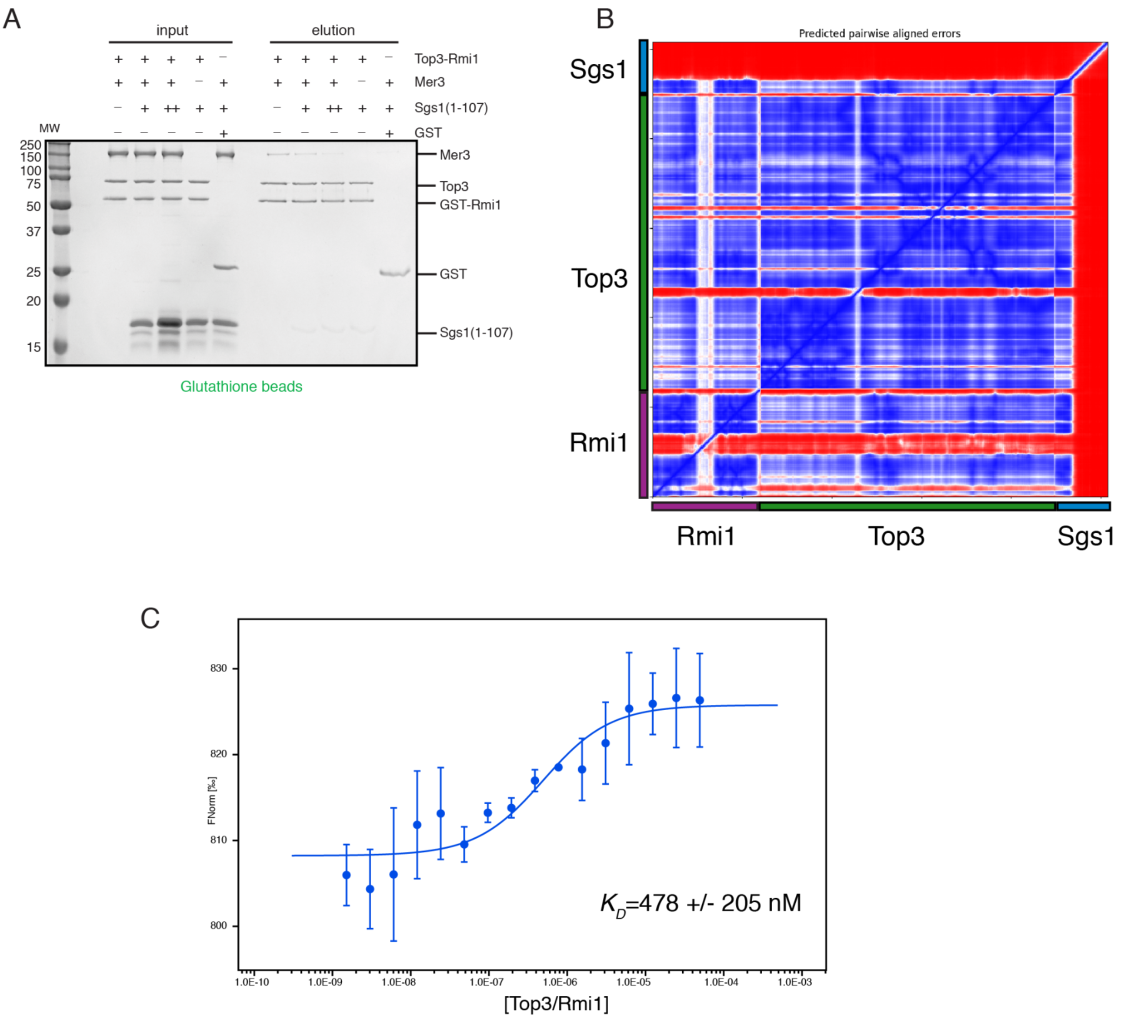

Supplementary Figure 6

Supplementary Figure 6

A) Glutathione pulldown of recombinant Top3/GST-Rmi1 (1 μM) against Mer3-Strep (1 μM), and in increasing concentrations of Sgs1$^{1\text{-}107}$ (8 or 25 μM). GST alone (3.7 μM) is used as a control for background binding.

B) PAE plot of the AF2 multimer predicted structure of Sgs1$^{1\text{-}107}$/Top3/Rmi1.

C) Microscale thermophoresis (MST) of Sgs1(1-605) binding to Top3/Rmi1 complex (blue). Sgs1(1-605) was fluorescently labelled and Top3/Rmi1 was titrated against Top3-Rmi1. Experiments were carried out in triplicate and the K_D of 478 nM was determined from the fitting curve in the NanoTemper Affinity Analysis v2.3 software (NanoTemper Technologies GmbH).

A

in vitro phosphosites

	log_2 intensities - dephospho			log2 intensities - purified			log2 intensities - okadaic				
Position	1	2	3	1	2	3	1	2	3	AA	Phospho (STY) Probabilities
80	24.730	NaN	NaN	23.805	24.344	NaN	28.902	29.160	29.127	S	GLQFFDKDDNFS(1)ITADDTQVTSK
958	NaN	NaN	NaN	30.347	30.288	30.111	32.286	32.397	32.191	S	KDLNS(1)LEK
398	25.878	25.742	26.115	26.132	25.872	25.112	29.142	29.399	29.152	S	FLLNNHIFS(1)K
17	29.719	29.962	30.104	31.762	31.697	32.094	33.811	33.854	34.384	S	SRPS(1)PNNIDFNDQSATFK
100	26.459	25.114	24.996	28.477	28.521	28.866	30.793	31.559	31.654	T	LFDHDLEQT(1)PDEEAKK
1092	25.968	24.226	23.756	23.203	22.671	22.585	34.330	34.635	34.729	T	NINT(0.004)T(0.996)PHLEK

B

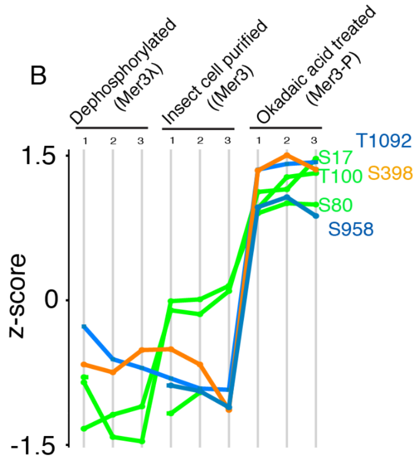

C

in vivo phosphosites

	log_2 intensities				
Position	1	2	3	Amino acid	Phospho (STY) Probabilities
80	23.50	24.30	NaN	S	DDNFS(1)ITADDTQVTSK
17	24.52	25.29	24.15	S	S(0.031)RPS(0.969)PNNIDFNDQSATFK
27	21.93	23.16	NaN	S	SRPSPNNIDFNDQS(0.893)AT(0.107)FK
100	22.11	23.83	NaN	T	LFDHDLEQT(1)PDEEAK

Supplementary Figure 7

Supplementary Figure 7 - Phosphoproteomics

A) Table of *in vitro* phosphosites showing the intensities of three replicate experiments for each of the three experimental conditions. Recombinant Mer3 treated with lambda-phosphatase (dephospho), untreated Mer3 purified from insect cells (purified) and Mer3 purified from insect cells treated with Okadaic acid. The lanes highlighted in green are common to our in vivo data (shown in C)

B) Profile plots of the z-transformed log2 intensities of the six in vitro phosphorylation sites measured on Mer3. Green trace is for the sites also found in our in vivo data. Blue trace is for those sites previously identified on Mer3 during meiosis (49). Orange shows the site only found in vitro.

C) Table of in vivo phosphosites, showing those sites identified in at least two of three replicate experiments. Those three sites highlighted in green were also found *in vitro* shown in A).

Supplementary Table 1. Plasmids used in the study.

Plasmid ID	Description	Reference
pWL413	pLIB-Mer3-Strep	This study
pWL493	pLIB-Mer3(1-1023)-Strep	This study
pWL522	pCOLI-Strep-Rfa1	This study
pWL526	pCDF-6xHis-Rfa2	This study
pWL527	pRSF-6xHis-Rfa3	This study
pWL746	pET11c-Dmc1	Lumir Krejci
pWL765	pLIB-GST-Rad54	This study
pWL808	pLIB-GST-Rmi1	This study
pWL812	pLIB-6xHis-Top3	This study
pWL856	pBIG1a-Top3/GST-Rmi1	This study
pWL910	PGK-Rad51	Lumir Krejci

pWL911	pRS416	Lumir Krejci
pWL993	pBIG1a-6xHis-MBP-Mlh1 6xHis-MBP-Mlh2	This study
pWL1645	pBIG1a-6xHis-Mlh1 Strep-Mlh2	This study
pWL1016	pLIB-6xHis-MBP-Sgs1-6xHis	This study
pWL1096	pLIB-6xHis-MBP-Sgs1-K706A-6xHis	This study
pWL1097	pLIB-Mer3(K167A)-Strep	This study
pWL1112	pLIB-Mer3(122-1023)-Strep	This study
pWL1758	pCOLI-6xHis-Sgs1(1-107)	This study
pWL1853	pLIB-Mer3(122-1187)-Strep	This study
pWL1897	pCOLI-6xHis-MBP-Sgs1(1-605)	This study
pWL1565	pGAD-C1	This study
pWL1564	pGBDU-C1	This study

pWL1700	pGAD-C1-Mer3	This study
pWL1716	pGBDU-C1-Mer3	This study
pWL1713	pGAD-C1-Mer3(1-1023)	This study
pWL1712	pGBDU-C1-Mer3(1-1023)	This study
pWL1826	pGAD-C1-Mer3(122-1187)	This study
pWL1827	pGBDU-C1-Mer3(122-1187)	This study
pWL1696	pGBDU-C1-Top3	This study
pWL1695	pGBDU-C1-Rmi1	This study

Supplementary Table 2. Oligonucleotides used in the study.

**Name**	**Sequence (5’-3’)**
oWL981	AAATCAATCTAAAGTATATATGAGTAAACTTGGTCTGACAGTTACCAATGC TTAATCAGTGAGGCACCTATCTCAGCGATCTGTCTATTT
1253 (5’-FAM)	TGGGTCAACGTGGGCAAAGATGTCCTAGCAATGTAATCGTCTATGACGT T
1253-T (5’-FAM)	GGGTCAACGTGGGCAAAGATGTCCTAGCAATGTAATCGTCTATGACGTT
1253C	AACGTCATAGACGATTACATTGCTAGGACATCTTTGCCCACGTTGACCCA

1254	TGCCGAATTCTACCAGTGCCAGTGATGGACATCTTTGCCCACGTTGACC C
1255	GTCGGATCCTCTAGACAGCTCCATGATCACTGGCACTGGTAGAATTCGG C
1256	CAACGTCATAGACGATTACATTGCTACATGGAGCTGTCTAGAGGATCCGA
315	AACGTCATAGACGATGATCCGATGCATATCCGCCTGCCCACGTTGACCC
320	GCGATAGTCTCTAGACAGCATGTCCTAGCAATACATTGCTAGGACATCTT
X12-3SC	TTGCTAGGACATGCTGTCTAGAGACTATCGC
3'overhang 25nt	GGACATCTTTGCCCACGTTGACCCA

Supplementary Table 3. Yeast strains used in this study.

Strain	Genotype	Source
yWL320	LP2749-9B	Lumir Krejci
yWL365	MATa, ura3-52, leu2-3, his3, trp1, gal4del, gal80del, GAL2-ADE2, LYS2::GAL1-HIS3, met2::GAL7-lacZ	Gerben Vader
yWL429	MAT a ho::LYS2 ura3 leu2::hisG trp1::hisG his3::hisG MAT alpha ho::LYS2 ura3 leu2::hisG trp1::hisG his3::hisG	Joao Matos
yWL430	MAT a ho::LYS2 ura3 leu2::hisG trp1::hisG his3::hisG MER3-9xMyc::KanMX4 MAT alpha ho::LYS2 ura3 leu2::hisG trp1::hisG his3::hisG MER3-9xMyc::KanMX4	This study

yWL444	MAT a ho::LYS2 ura3 leu2::hisG trp1::hisG his3::hisG MER3-9xMyc::KanMX4 Top3-3HA::hphNTI MAT alpha ho::LYS2 ura3 leu2::hisG trp1::hisG his3::hisG MER3-9xMyc::KanMX4 Top3-3HA::hphNTI	This study
